# Structure-informed functional connectivity driven by identifiable and state-specific control regions

**DOI:** 10.1162/netn_a_00192

**Published:** 2021-06-21

**Authors:** Benjamin Chiêm, Frédéric Crevecoeur, Jean-Charles Delvenne

**Affiliations:** Institute of Communication Technologies, Electronics, and Applied Mathematics, Department of Mathematical Engineering, Université Catholique de Louvain, Louvain-la-Neuve, Belgium; Institute of Neuroscience, Division of Systems and Cognitive Neuroscience, Université Catholique de Louvain, Brussels, Belgium; Institute of Communication Technologies, Electronics, and Applied Mathematics, Department of Mathematical Engineering, Université Catholique de Louvain, Louvain-la-Neuve, Belgium; Institute of Neuroscience, Division of Systems and Cognitive Neuroscience, Université Catholique de Louvain, Brussels, Belgium; Institute of Communication Technologies, Electronics, and Applied Mathematics, Department of Mathematical Engineering, Université Catholique de Louvain, Louvain-la-Neuve, Belgium

**Keywords:** Connectome, Structure, Function, Controllability, Control regions

## Abstract

Describing how the brain anatomical wiring contributes to the emergence of coordinated neural activity underlying complex behavior remains challenging. Indeed, patterns of remote coactivations that adjust with the ongoing task-demand do not systematically match direct, static anatomical links. Here, we propose that observed coactivation patterns, known as functional connectivity (FC), can be explained by a controllable linear diffusion dynamics defined on the brain architecture. Our model, termed *structure-informed* FC, is based on the hypothesis that different sets of brain regions controlling the information flow on the anatomical wiring produce state-specific functional patterns. We thus introduce a principled framework for the identification of potential control centers in the brain. We find that well-defined, sparse, and robust sets of control regions, partially overlapping across several tasks and resting state, produce FC patterns comparable to empirical ones. Our findings suggest that controllability is a fundamental feature allowing the brain to reach different states.

## Author Summary

Understanding how brain anatomy promotes particular patterns of coactivations among neural regions is a key challenge in neuroscience. This challenge can be addressed using network science and systems theory. Here, we propose that coactivations result from the diffusion of information through the network of anatomical links connecting brain regions, with certain regions controlling the dynamics. We translate this hypothesis into a model called *structure-informed functional connectivity*, and we introduce a framework for identifying control regions based on empirical data. We find that our model produces coactivation patterns comparable to empirical ones, and that distinct sets of control regions are associated with different functional states. These findings suggest that controllability is an important feature allowing the brain to reach different states.

## INTRODUCTION

Recently, approaches combining magnetic resonance imaging (MRI) and network science emerged in order to characterize links among neural regions of interest (ROIs; Bassett & Sporns, 2017). Most studies focus either on structural connections or on functional interactions, which capture two distinct aspects of brain connectivity. On the one hand, diffusion MRI (dMRI) with tractography (Mori & Zhang, 2006) enables the mapping of white matter pathways and describes the anatomical links between ROIs. This structural description of the human brain forms a network called the *connectome* (Hagmann et al., 2008; Sporns, Tononi, & Kötter, 2005). On the other hand, the blood-oxygenation-level dependent (BOLD) signal in functional MRI (fMRI) provides an estimate of brain activity in gray matter areas (Ogawa, Lee, Kay, & Tank, 1990). The matrix of pairwise Pearson’s correlation coefficients between regional BOLD time series is a common tool to quantify *functional connectivity* (FC) among ROIs (Bassett & Sporns, 2017). Unlike the connectome, functional connectivity varies over short timescales and across resting-state and task conditions (Cole, Bassett, Power, Braver, & Petersen, 2014).

An important challenge in neuroscience is to characterize the relationship between the connectome and functional connectivity (Batista-García-Ramó & Fernández-Verdecia, 2018; C. J. Honey, Thivierge, & Sporns, 2010; Suárez, Markello, Betzel, & Misic, 2020). Several approaches have been proposed in the literature in order to elucidate this relationship in macroscale brain networks and understand the importance of the anatomical organization in promoting particular patterns of activity. Along with methods based on graph signal processing and spectral decompositions (Preti & Van De Ville, 2019; Tewarie et al., 2020), it has been proposed that describing the link between the connectome and FC requires a model of information flow between ROIs (Avena-Koenigsberger, Misic, & Sporns, 2018). For instance, models based on random walks and diffusion on the connectome have been able to partly reproduce resting-state FC (Abdelnour, Voss, & Raj, 2014; Goñi et al., 2014; Mišić et al., 2015). Viewing the brain as a dynamical system allows us to study the controllability of this system, that is, its ability to account for context-dependent control signals in order to affect the overall state of the brain (Gu et al., 2015; Medaglia, 2019). The framework of network controllability requires the definition of input regions, that is, ROIs capable of integrating control signals (Liu, Slotine, & Barabási, 2011). Earlier work demonstrated that any single input region was theoretically sufficient to get controllability of the connectome (Gu et al., 2015; Pasqualetti, Gu, & Bassett, 2019; Tu et al., 2018). One shortcoming is that although controllable in theory, some configurations are practically unfeasible as they would require excessive control energy. Moreover, several pieces of evidence from the fields of motor and cognitive control suggest that sets of regions are responsible for the control of brain activity (Cole et al., 2014; Dosenbach et al., 2007; Eisenreich, Akaishi, & Hayden, 2017; Omrani, Murnaghan, Pruszynski, & Scott, 2016; Power & Petersen, 2013). Despite these advances in brain communication modeling and connectome controllability, an integrated explanation for the emergence of multiple FC patterns from the static structure of the connectome is still lacking.

Here, we develop a principled approach modeling state-specific FC on the connectome. We leverage the observation that the Gramian matrix used in controllability studies (Gu et al., 2015; Pasqualetti, Zampieri, & Bullo, 2014) corresponds to the covariance matrix of the activities in the different nodes of a network, assuming a linear transition dynamics among them. This observation brings us to introduce the concept of structure-informed FC, that is, the pairwise functional correlation matrix derived from the structure of the connectome. Since this matrix depends on the choice of input nodes, we show that it is possible to identify the set of input ROIs maximizing the mapping between structure-informed and empirical FC in different states. Using dMRI and fMRI data (resting-state and seven tasks) from the Human Connectome Project (Van Essen et al., 2013), we find that sparse input sets produce FC matrices that are comparable to empirical ones. Moreover, we show that the identified sets are well defined, stable, and state-specific. We discuss their properties and the fact that the method is able to capture the singularity of resting-state compared with the other task-related conditions. Overall, our approach relies on a model linking structure and function in brain networks in order to identify possible subsets of brain regions underlying task-specific control.

## RESULTS

### Structure-Informed Functional Connectivity

In order to investigate how the connectome shapes functional connectivity (FC), we study the covariance matrix of a linear dynamics defined on the connectome. In a network of *n* nodes, let **x**(*k*) be the *n*-dimensional state-vector containing the activity level of each node at time *k*. The trajectory of **x** is governed by the following equation:

$$x\left(k+1\right)=Ax\left(k\right)+Bu\left(k\right).$$ (1)

Here, the *n* × *n* system matrix **A** describes the interactions among the nodes of the network, the columns of the *n* × *m* input matrix **B** are canonical vectors identifying the *m* input nodes, and **u**(*k*) is an *m*-dimensional vector providing the value of external input signals at time *k*.

When the inputs to the system, that is, the signals in **u**, are white noise signals, it can be shown that the steady-state covariance matrix of the states **Σ** = Cov(**x**) satisfies the following Lyapunov equation (see Methods for the derivation):

$$\Sigma ={A\Sigma A}^{T}+{BB}^{T}.$$ (2)

**Figure 1.**
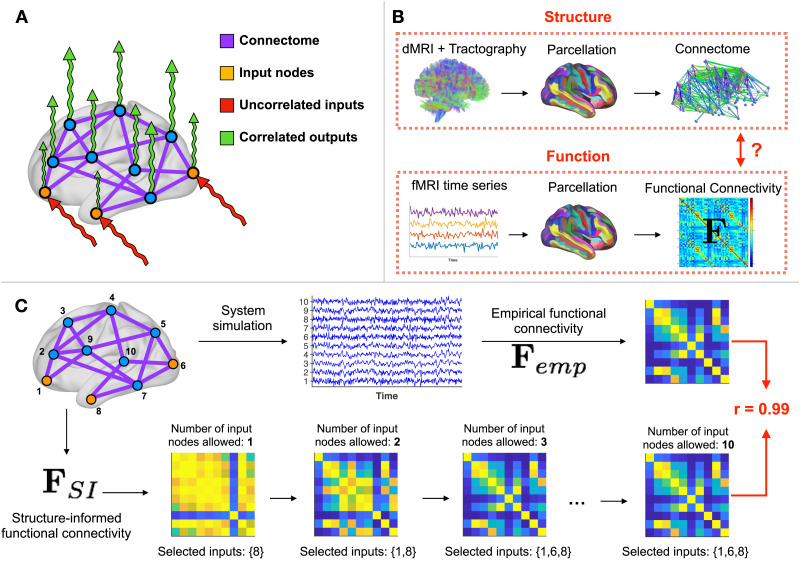
Overview of the approach. (**A**) In order to investigate how the connectome shapes functional connectivity, we define a diffusion dynamics on the connectome (purple) and excite it with uncorrelated signals (white noise, red). Depending on the set of input nodes (orange) driving the dynamics, the output signals (green) present correlation patterns that are similar to empirical data. (**B**) Data-processing workflow. (Top row) We extract the connectome using diffusion imaging (dMRI) and tractography. Nodes correspond to ROIs from a predefined automatic parcellation. (Bottom row) At each ROI, we also retrieve the fMRI BOLD time series and compute the functional connectivity matrix **F**_*emp*_ between these signals. This step is repeated for seven tasks and resting-state. (**C**) Example on simulated data. We start from a network of *n* = 10 nodes, with uniformly distributed random weights on the edges. (Top row) We choose a set of *m* = 3 input nodes, simulate the noise diffusion process, and compute the empirical functional connectivity matrix **F**_*emp*_. (Bottom row) Our framework applied to the network identifies the correct set of input nodes and generates a structure-informed functional connectivity matrix **F**_*SI*_ comparable to the empirical one.

Here, we see that the solution **Σ** depends on the structure of the network and the dynamical model through the system matrix **A**, and on the set of input nodes defined by **B** (Figure 1A). The solution to Equation 2 is known as the controllability Gramian. Here, in contrast to previous studies where **Σ** is used to derive quantitative control properties of individual nodes in the network (Gu et al., 2015; Karrer et al., 2020; Pasqualetti et al., 2014), we interpret the Gramian as the state-covariance matrix obtained by stochastic excitation of the system through a set of control nodes. This allows us to relate it to the concept of functional connectivity. Indeed, after variance normalization, **Σ** becomes a correlation matrix $\tilde{\Sigma }$ (see Methods) and constitutes the FC matrix associated with the network and its dynamics, which we term the *structure-informed* FC and denote **F**_*SI*_:

$$F_{SI}=\tilde{\Sigma }.$$ (3)

Using the mathematical relation between the network structure and the correlation matrix of the system, we turn to the problem of identifying the set of control inputs defined by **B**, given an empirical FC matrix **F**_*emp*_ obtained from external recordings of the system. For that, we formulate the optimization problem

$$\begin{array}{c} \;B^{*}=\arg\;\max_{B}\;sim\left(F_{SI},F_{emp}\right)\\ \text{such}\;\text{that}\;m\leq U, \end{array}$$ (4)

where **F**_*SI*_ is a function of **B**; *m* is the number of columns of **B**, that is, the cardinality of the input set; and *U* is an upper bound to be fixed in order to control the number of input nodes.

In the present work, we apply this approach to connectomes and FC matrices extracted from MRI data (Figure 1B). We consider a diffusion dynamics to model interactions among ROIs in the connectome (see Methods) as suggested in previous studies on large-scale brain communication (Abdelnour et al., 2014; Avena-Koenigsberger et al., 2018). The similarity between **F**_*SI*_ and **F**_*emp*_ is the correlation score (see Methods). For the optimization, we use a heuristic approach (genetic algorithm, see Methods). To mitigate the lack of optimality guarantee, we run the algorithm multiple times and denote the set of ROIs consistently selected across runs as the consensus input set (see Methods).

We provide an illustration of the method based on simulated data in Figure 1C. First, we simulate 2,000 time steps of a diffusion process driven by white noise on a graph composed of *n* = 10 nodes (*m* = 3 input nodes), with edge weights uniformly distributed between 0 and 1. Using these time series, we compute the associated FC matrix **F**_*emp*_. Then, we solve Problem 4 for *U* varying from 1 to *n*. We observe that the method retrieves the correct input set and produces an FC matrix that is similar to the empirical one.

### Linking the Connectome to Multiple Functional States

We apply our approach and solve Problem 4 with empirical MRI data of 100 unrelated individuals. For each individual, we extract a connectome and FC matrices for resting-state and seven tasks (Figure 1B; see Methods for a description of the tasks). Although the properties of resting-state FC are known to be fundamentally different from that of task FC (Deco, Jirsa, & McIntosh, 2011), we deliberately choose to treat resting-state in the same way as task conditions in order to test whether our approach is able to distinguish it. For simplicity, we refer to both resting-state and task conditions as *states* in the remainder of the manuscript. Finally, we use the brain parcellation introduced by Destrieux, Fischl, Dale, and Halgren (2010) and composed of *n* = 164 ROIs including subcortical structures and cerebellum.

**Figure 2.**
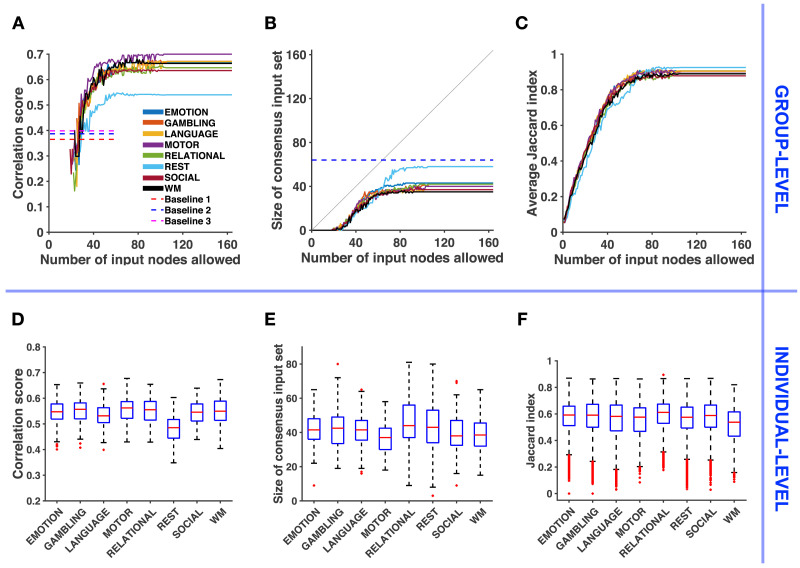
Relating structure-informed and empirical functional connectivity: Group-level analysis and individual-level variability. (**A**) Correlation score between structure-informed and empirical functional connectivity with respect to the number of input nodes allowed *U* (group level). **F**_*SI*_ is obtained using the consensus input set. Dashed lines represent baselines corresponding to the similarity between **F**_*emp*_ and (1) the adjacency matrix of the connectome, (2) **F**_*SI*_ based on a relabeled connectome and (3) **F**_*SI*_ obtained with a random input set (see Methods). (**B**) Size of the consensus input set with respect to the number of input nodes allowed *U* (group level). The gray line denotes the identity function *y* = *x*. The dashed blue line corresponds to the minimum number of input nodes selected for Baseline 2, over all conditions and all randomizations. (**C**) Average Jaccard index between the 30 input sets identified by the optimization algorithm with respect to the number of input nodes allowed *U* (group level). (**D**) Variability across individuals of the correlation score between structure-informed and empirical correlation matrices, with *U* = *n*. (**E**) Variability of the size of the corresponding consensus input set. (**F**) Jaccard index between all pairs (*N* = 4,950) of consensus input sets (25 selections over 30 runs) across individuals.

For the group-level analysis, we compute an average connectome and an average FC matrix **F**_*emp*_ for each state (see Methods and Supplementary Figure S1). In order to study the stability of our results with respect to the number of input ROIs, we solve Problem 4 with *U* increasing from 1 to *n*. For each upper bound *U*, we define the consensus input set as the set of ROIs selected at least 25 times over 30 optimization runs (see Methods). Figure 2A shows the correlation score *r* between **F**_*emp*_ and **F**_*SI*_ using the consensus input set. The curves increase with *U*, up to small drops due to the heuristic nature of the optimization (see Methods), until they reach a plateau at values ranging from *r* = 0.54 for resting-state to *r* = 0.7 for the motor task. We can compare these values with three baselines (see Methods for details about the baseline definitions). The first one is the correlation score between **F**_*emp*_ and the adjacency matrix of the connectome. The second is the plateau correlation obtained by applying our approach to a randomly relabeled connectome. The third baseline is the maximum correlation score between **F**_*SI*_ and **F**_*emp*_ obtained with random input sets having the same average cardinality as the identified sets. In Figure 2A, we draw for each baseline the highest value across states and see that our approach produces a better matching for all states. Figure 2B shows that the consensus input set is empty for all states until we allow the selection of at least 19 input nodes. Then its size stabilizes between *m* = 35 for the working-memory task (WM) and *m* = 58 for resting-state. These values are lower than the number of input nodes selected when applying our approach to a randomly relabeled connectome (Baseline 2, *m* = 64, minimum across states and randomizations). To evaluate the consistency of identified input sets across optimization runs, we report in Figure 2C the evolution of the average Jaccard index $\overset{ˉ}{J}$ (see Methods), computed over all pairs of the 30 optimized input sets. We observe that the method selects consistent input sets ($\overset{ˉ}{J}$ ≥ 0.85) when *U* ≥ 70.

We also perform an individual-level analysis in the following way. As the plateaus observed in Figures 2A and 2B are also observed for a random sample of 20 individuals (see Supplementary Figures S5 and S6), we apply the method to each individual and set *U* = *n* in order to reduce the computational cost of the optimization. We obtain one consensus input set and one correlation score for each individual and for each state. In Figure 2D, we notice that the correlation scores are lower than at the group level, for all states. A repeated-measures ANOVA determines that the mean correlation score differs significantly between states (*F*(7, 693) = 27.928, *p* < 10^−25^, Greenhouse-Geisser corrected). The variance in each condition does not significantly differ (Levene’s test, *p* > 0.5), and a post hoc analysis after visual inspection confirms that the mean correlation score in resting-state is significantly different than in any task condition (Tukey’s HSD, *p* < 0.005). The post hoc analysis also reveals that the mean correlation score significantly differs between the language task and the motor task (Tukey’s HSD, *p* < 0.005). Figure 2E shows the variability of the size of the consensus input set in the population. The relational processing task and the resting-state display a higher variability in the number of input ROIs selected than other states. In Figure 2F, we evaluate the variability of the consensus input set *in the population* by computing the Jaccard index *J* of all pairs of the 100 consensus input sets (one for each individual). We observe a moderate overlap of the consensus input set across individuals (median *J* ≈ 0.6, expected value of *J* for randomly chosen sets with cardinality *m* = 40 : 𝔼{*J*} ≈ 0.14; see Methods for the derivation). A Friedman test finds that Jaccard indices come from different distributions across tasks (*p* < 10^−244^).

### Analysis of Input ROIs Across Functional Subsystems

**Figure 3.**
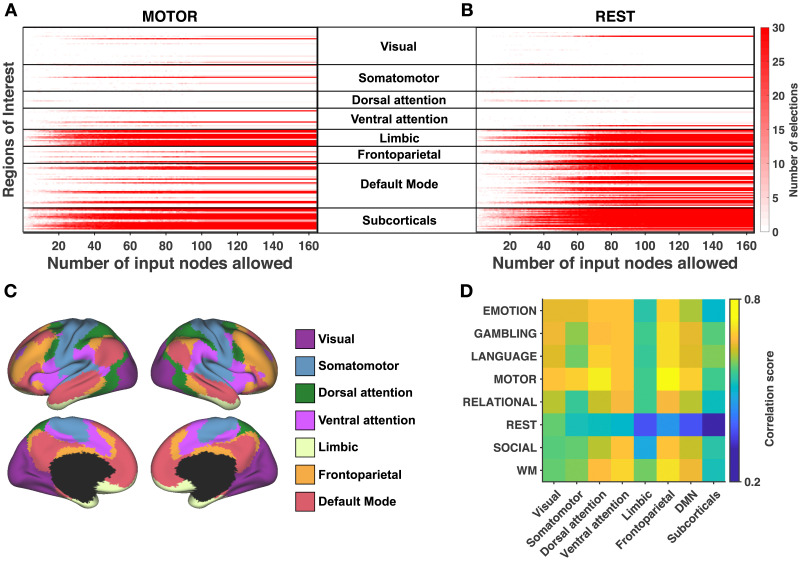
Analysis across functional subsystems (group level). (**A**) (resp. **B**) Evolution of the number of selections (from 0-white, to 30-red) of each ROI with respect to the number of input nodes allowed *U* for the motor task (resp. for resting-state). ROIs are arranged according to the functional subsystems described by Yeo and colleagues (Yeo et al., 2011). The cerebellum is included in the “subcorticals” subsystem for visualization and corresponds to the last two lines (left and right hemispheres). Corresponding figures for the other tasks are available in Supplementary Figure S7. (**C**) Cortical localization of Yeo’s subsystems. (**D**) Correlation between structure-informed and empirical functional connectivity with *U* = *n*, split into Yeo’s subsystems. Structure-informed functional connectivity is computed using the consensus input set.

At the group level, we turn our attention to the ROIs composing the input sets that we identified. In Figures 3A (motor task) and 3B (resting-state), we can follow the evolution of the number of selections of each ROI when the maximum cardinality *U* of the input set increases. We point out that when *U* is incremented, we perform the new optimization runs while ignoring previously computed solutions in order to assess the consistency of successively computed solutions. A first observation is that the selection of input ROIs is stable, that is, once a region is selected it is typically selected again for higher values of *U*, as indicated by the horizontal red lines. Moreover, dark red pixels for a given ROI indicate that it is consistently selected across 30 independent optimization runs for a fixed *U*. We make a second observation by grouping ROIs according to the functional **subsystems** defined by Yeo et al. (2011) and presented in Figure 3C (we include the cerebellum in the “subcortical” subsystem for visualization). Regions belonging to limbic and subcortical subsystems are selected together, up to some exceptions. These observations are also valid for the other tasks (corresponding figures are available in Supplementary Figure S7).

Recent studies investigated how the connectome shapes functional connectivity at the level of subsystems and showed that the coupling between structure and function is stronger for some subsystems than for others (Baum et al., 2020; Mišić et al., 2016; Osmanlıoğlu et al., 2019; Tipnis, Amico, Ventresca, & Goni, 2018; Vázquez-Rodríguez et al., 2019). In Figure 3D, we use the consensus input sets identified at the group level and compute the correlation score between the entries of **F**_*SI*_ and **F**_*emp*_ associated with the subsystems of Figure 3C (see Supplementary Note 4). The results indicate that the association is the greatest in the frontoparietal lobe during the motor task (*r* = 0.74). Moreover, the limbic and subcortical subsystems show low correlation scores for all states, while we observe for the resting-state a gradient going from high correlation in primary sensory systems (visual, somatomotor) to low correlation in systems associated with higher order cognition (limbic, default mode, subcorticals).

### Analysis of Input ROIs Across States

Next, we compare the composition of the identified input sets across states, at the group level. Since we observed in Figure 2A that the correlation score reaches a plateau when *U* increases, we set *U* = *n* for this analysis. Moreover, we increase the number of optimization runs to 100 to evaluate more precisely the selection of each ROI. Thus, we obtain 100 input sets for each condition.

**Figure 4.**
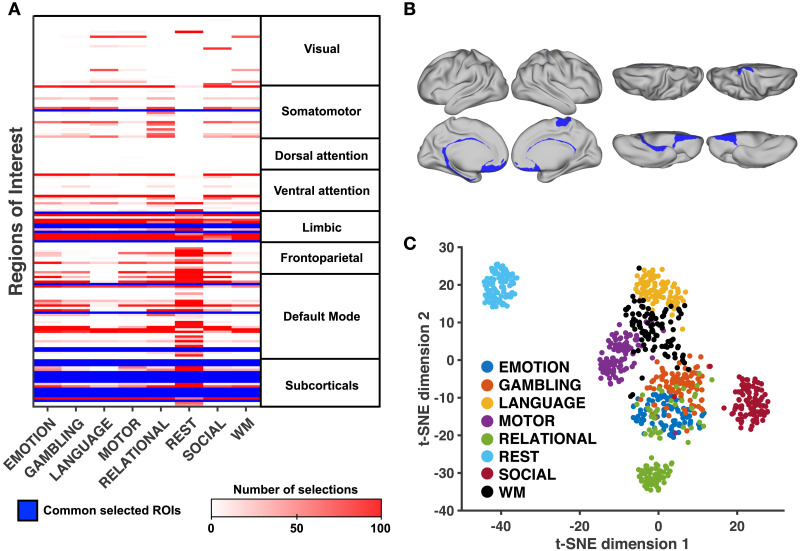
Analysis across functional states. (**A**) Table summarizing the most frequently selected ROIs for each task. ROIs that are consistently selected at least 90 times over 100 runs for all functional states are highlighted in blue. ROIs are grouped according to Yeo’s functional subsystems (Yeo et al., 2011). The cerebellum is included in the “subcorticals” subsystem for visualization and corresponds to the last two lines (left and right hemispheres) (**B**) Cortical view of ROIs consistently selected across all tasks and resting-state. (**C**) Two-dimensional projection of all input sets (100 runs, eight states). We use the *t*-distributed stochastic neighbor embedding algorithm (*t*-SNE, see Methods) in order to visualize the Jaccard similarity among all input sets. Each data point represents one such input set, and their proximity is proportional to their similarity.

Figure 4A depicts the number of selections of each ROI across states. Blue lines indicate ROIs that have been selected at least 90 times for all states. These ROIs mostly correspond to subcorticals (accumbens nucleus, amygdala, hippocampus, pallidum, thalamus, and subcallosal gyrus) and limbic regions (medial orbital sulcus, gyrus rectus, and left suborbital sulcus). Regions of the default mode network (pericallosal sulcus, right suborbital sulcus and left posterior-ventral part of the cingulate gyrus) and of the somatomotor system (right paracentral lobule) complete the set of ROIs consistently selected across states. A cortical view of these regions is shown in Figure 4B. We observe that most of these regions are located in the midline. Supplementary Table S17 provides the detailed numerical results by ROI.

**Figure 5.**
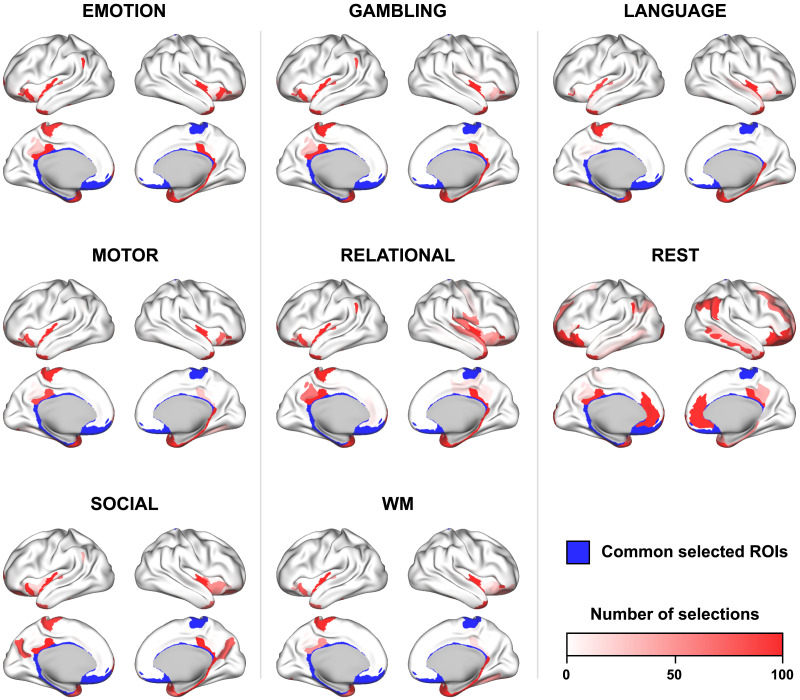
Cortical surface view of state-specific input ROIs. Over 100 runs of the optimization algorithm with *U* = *n*, we depict for each state the number of times each ROI is selected. Regions that were consistently selected across all states (≥90 selections) are shown in blue. Detailed numerical results by ROI are available in Supplementary Table S17.

In order to visualize the divergence of input sets across states, we use a dimensionality reduction method to project in two dimensions the *n*-dimensional binary vectors indicating which ROIs belong to each input set (*t*-distributed stochastic neighbor embedding; see Methods). In Figure 4C, each data point represents one identified input set (100 runs, 8 states), and the proximity with each other is indicative of their overlap (Jaccard similarity). We distinguish clusters of points corresponding to different states. In particular, the cluster corresponding to resting-state is isolated. Among task conditions, there is a partial overlap of the clusters, with the input sets related to the social cognition task being more isolated from the others. A comparative cortical view of input ROIs for each condition is provided in Figure 5.

### Topological Properties of Input ROIs

In order to gain further insight into the topological properties of input ROIs in the connectome, we analyze the statistical association of the number of selections of each ROI with two nodal metrics: the weighted degree and modal controllability. The weighted degree of a node describes the strength of the connections with its neighbors, while modal controllability describes the ability of a node to drive the network towards hard-to-reach states requiring much control energy (see Methods and references Gu et al. [2015] and Pasqualetti et al. [2014] for further details about modal controllability). For all tasks and resting-state, we report in Table 1 the correlation between these nodal metrics and the number of selections of ROIs. On the one hand, we find an inverse relationship between weighted degree and number of selections (lowest association: Spearman’s *ρ* = −0.3963 in resting-state, *p* < 10^−7^ for all states), which suggests that low-degree ROIs are selected more often. On the other hand, we find a direct relationship between modal controllability and number of selections (lowest association: Spearman’s *ρ* = 0.4769 in resting-state, *p* < 10^−10^ for all states), indicating that ROIs having a high modal controllability are selected more frequently. While both associations are significant, they are not absolute and they do not prevent the selection of high-degree ROIs such as subcortical structures (see Supplementary Figure S1).

**Table 1.** Spearman’s rank correlation between the number of selections of each ROI (out of 100 independent optimization runs, with *U* = *n*, group-level) and two nodal coefficients: the strength (weighted degree) and the modal controllability (Pasqualetti et al., 2014). EMO: emotional processing, GAM: gambling, LAN: language processing, MOT: motor task, REL: relational processing, REST: resting-state, SOC: social cognition, WM: working-memory.

Spearman’s *ρ*	EMO	GAM	LAN	MOT	REL	REST	SOC	WM
Weighted degree	−0.5190	−0.4873	−0.4937	−0.4621	−0.4866	−0.3963	−0.4415	−0.5014
Modal control	0.6149	0.5767	0.5672	0.5517	0.5803	0.4769	0.5305	0.5801

We also compare the modal controllability and weighted degree of cortical regions consistently selected across states (in blue, Figures 4B and 5) with that of other regions. We find that the modal controllability is higher (Wilcoxon rank-sum test, *p* < 0.001) and the weighted degree is lower (Wilcoxon rank-sum test, *p* < 0.01) in these regions.

### Robustness of Consensus Input Sets

Finally, we study the robustness of the link between structure-informed (**F**_*SI*_) and empirical (**F**_*emp*_) functional connectivity when the consensus input set is attacked. Here, an attack refers to the removal of an ROI from the initial input set, not from the connectome. We start from the correlations between **F**_*SI*_ and **F**_*emp*_ obtained at the group level with *U* = *n* (Figure 2A). We progressively remove nodes from the consensus input set until it becomes empty. After each removal, we compute the correlation score obtained with the attacked input set. Since we previously observed that low-degree (resp. high modal controllability) ROIs are more likely to be part of the input set, the removal ordering is fixed by increasing order of weighted degree (resp. by decreasing order of modal controllability). In addition, we report the results related to 50 random removal sequences.

**Figure 6.**
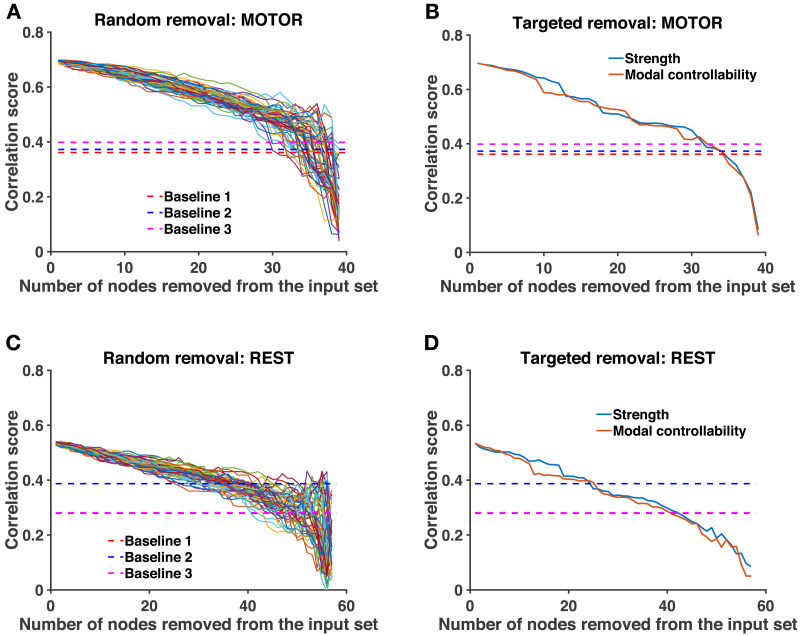
Robustness analysis. (**A**) (resp. **C**) Evolution of the correlation between structure-informed **F**_*SI*_ and empirical functional connectivity **F**_*emp*_ as a function of the number of ROIs removed from the consensus input set. Dashed lines represent the three baselines (see Methods), that is, the correlation between **F**_*emp*_ and (a) the adjacency matrix of the connectome, (b) **F**_*SI*_ based on a relabeled connectome, and (c) **F**_*SI*_ obtained with a random input set. We consider 50 random removal orderings. (**B**) (resp. **D**) Same analysis, with removal ordering fixed by increasing weighted degree and decreasing modal controllability.

In Figure 6, we show the results for the motor task and the resting-state. We observe that the correlation score between **F**_*SI*_ and **F**_*emp*_ decreases slowly with the number of nodes removed from the consensus input set, no matter the removal ordering. For the motor task (resp. for resting-state), up to 75% (resp. 40%) of the nodes can be removed from the consensus input set before we reach correlation scores comparable to the three baselines previously defined (see Methods). Similar observations are valid for the other tasks (see Supplementary Figures S9 and S10).

## DISCUSSION

In this work, we studied the structure-function relationship in brain networks (Batista-García-Ramó & Fernández-Verdecia, 2018; C. J. Honey et al., 2010; Suárez et al., 2020) across different task conditions as well as in resting-state. We showed that functional connectivity (FC), that is, the coactivations among brain regions, can be explained by the correlations between the activities of these regions resulting from a linear dynamics spreading through the structure of the brain. This model, termed *structure-informed* FC, happens to be mathematically linked to the Gramian matrix used in controllability studies (Gu et al., 2015; Karrer et al., 2020; Pasqualetti et al., 2014). This provides a novel interpretation of FC in which we can leverage control theory to explain state-specific FC configurations arising from a fixed anatomical architecture. We thus proposed that different groups of regions controlling a diffusion dynamics through the wiring diagram of the brain are responsible for FC matrices corresponding to different states. We introduced a principled approach to test this hypothesis and found that sparse and stable groups of control regions, which partially overlap across states, generate FC matrices that are statistically comparable to empirical ones.

### Combining Brain Communication Models and Linear Controllability

Several models of brain communication dynamics have been proposed in order to map structure and function during resting-state and in the absence of external input signals (Avena-Koenigsberger et al., 2018; Avena-Koenigsberger et al., 2019; Goñi et al., 2014; Seguin, van den Heuvel, & Zalesky, 2018). In parallel, other linear models considered the idea that brain activity can be modulated by external signals and first assumed that all brain regions are input nodes (Galán, 2008; C. Honey et al., 2009). Subsequent studies on the controllability of brain networks (Medaglia, 2019) relaxed this assumption and assessed the role of individual input nodes (Gu et al., 2015; Karrer et al., 2020). Later, the study of brain state transitions pointed to the theoretical and empirical motivations of considering a set of control regions (Gu et al., 2017). However, the question of how to identify the control set associated with a given brain state from empirical data remains challenging (see Supplementary Note 1 for a comparison with previous work). Here, we addressed this challenge and proposed a principled method to identify state-specific sets of control regions from empirical data. We considered a symmetric Laplacian diffusion dynamics following previous work (Abdelnour et al., 2014), though the framework can be applied to other communication dynamics. For instance, we also tested our approach with (a) the random-walk Laplacian instead of the symmetric normalized version or (b) with the adjacency matrix of the connectome as the transition matrix. We obtained similar results with significant overlap of the computed inputs sets (see Supplementary Table S16 for a comparison). Alternative dynamics including decentralized (i.e., directed) brain communication models (Goñi et al., 2014; Seguin et al., 2018) could provide complementary insights into the structure-function relationship in the human brain.

As in former connectomic studies (Abdelnour et al., 2014; Gu et al., 2015; Karrer et al., 2020; Pasqualetti et al., 2014), our approach relies on linear time-invariant modeling (Equation 1). Despite the known nonlinearities of neural dynamics (Breakspear, 2017), first-order approximations have been proven useful in capturing various aspects of brain functioning at different spatiotemporal scales (Galán, 2008; Schaub, Billeh, Anastassiou, Koch, & Barahona, 2015; Yan et al., 2017). In addition, time-invariance implies that the structure of the system does not evolve over time. Although the white matter architecture evolves over long timescales (Sexton et al., 2014; Tang et al., 2017), significant changes in the topology of the connectome are not expected over the duration of an MRI scan. Assuming linearity and time-invariance allowed us to derive an analytical expression of structure-informed FC (Equation 3). Since the heuristic optimization computes this matrix a large number of times in order to find a near-optimal input set, relying on an efficiently solved analytical expression of structure-informed FC rather than simulating the system at each iteration is computationally beneficial, although the computational cost remains a limitation of our framework. In sum, we argue that linear and time-invariant modeling of functional connectivity constitutes a reasonable and computationally tractable approach. Future studies are required to assess how much these assumptions can or should be relaxed in light of more realistic models compatible with the biology.

We considered in this work a coarse-grained parcellation spanning the entire brain (Destrieux et al., 2010). We suggest that the proposed method is also suitable at the level of subregions. For example, future research could investigate control centers in the cerebellum, using a refined and dedicated parcellation of cerebellar nuclei (Diedrichsen et al., 2011). At the microscale, studies of the controllability of the *C. elegans* connectome have shown the potential of linear models at the neuronal level (Towlson & Barabási, 2020; Yan et al., 2017). It has also been reported that the strength of the structure-function relationship in brain networks is parcellation-dependent (Messé, 2020). Here, the application of our approach with a coarser whole-brain parcellation (Desikan-Killiany atlas, 84 ROIs, see Supplementary Figure S11) produced lower correlation scores but did not invalidate our conclusions. We encourage future research assessing the relevance of the proposed model across parcellations spanning multiple spatial scales.

### Well-Defined Sets of Control Regions Drive State-Specific Functional Connectivity

In our analyses, we identified sparse groups of regions that are thought to support the control of state-specific brain activity. The fact that our method finds such *sparse* input sets (Figure 2B), that is, that empirical FC can be explained more simply from the true connectome structure than from a randomly relabeled network, suggests the fitness of our model, in line with Occam’s razor principle. Our model also captured differences between states in terms of their respective input sets (Figure 4C), supporting the idea that different states are triggered by partially overlapping yet distinct sets of control regions. Because our approach involves a heuristic optimization algorithm, we assessed the consistency of the identification procedure (Figure 3A–B) and the robustness of the identified input sets (Figure 6). The slow decrease of the correlation score observed when the optimal input set is eroded, no matter the removal ordering, indicates a redundant and collective effect of the control regions. Moreover, we found that ROIs having low degree and high modal controllability, which are topological properties associated with the brain structure independently of any activation measure, have a higher probability to be part of an input set (Table 1). Still, we showed that the selection of control regions was not exclusively driven by those nodal properties of the connectome, since subcortical areas are high-degree nodes consistently selected across states (Figure 4A and Supplementary Figure S1) and input sets are specific to each functional state (Figure 4C). Together, these results suggest that the identified input ROIs play a central role in driving FC across the white matter wiring.

Importantly, this role does not imply that identified ROIs systematically match the active areas traditionally detected in fMRI analyses. For example, the primary motor cortex (M1) is not part of the input set of the motor task (Figure 5), although it displays strong activation in the functional data. This activation results from the fact that M1 forms a hub in the motor task, receiving projections from multiple regions, including the somatosensory and parietal cortices as well as premotor areas, and sending output commands to the periphery. This “centrality” however does not entail that M1 is part of the set of drivers that put the brain in a state that is suitable for motor control. In this regard, our findings are supported by recent experimental evidence in mice showing that thalamic inputs are essential to drive the motor cortex during movement execution (Sauerbrei et al., 2020). A similar example is that of Wernicke’s area, which was not part of the input set of the language processing task (Figure 5) but whose activation is often associated with language understanding. More generally, the fact that drivers are preferentially (but not exclusively) ROIs with low degree and high modal controllability is consistent with the idea that reaching demanding states requires the control of decentralized and distributed areas, which in turn influence the whole system including hubs, such as M1 or Wernicke’s area (Amico, Arenas, & Goñi, 2019; Eisenreich et al., 2017; Gu et al., 2015; Liu et al., 2011; Omrani et al., 2016).

In order to gain a better insight into the role of the ROIs that we identified, we turn our attention to the drivers common to all states. The presence of subcortical structures (including basal ganglia, amygdala, hippocampus, and thalamus) in the input set of all states is consistent with their strong contribution to whole-brain communication (Bell & Shine, 2016), motor control (Shadmehr & Krakauer, 2008), language processing (Ketteler, Kastrau, Vohn, & Huber, 2008), reward-related processing (Delgado, Nystrom, Fissell, Noll, & Fiez, 2000) and cognition in general (Koziol & Budding, 2009). Anatomical and physiological evidence established the existence of cortico-subcortical loops supporting functionally segregated systems (Alexander, DeLong, & Strick, 1986). Within these loops, which include the anterior cingulate and dorsolateral prefrontal cortices that have been designated as cognitive control centers (Cole et al., 2013; Dosenbach et al., 2007; Power & Petersen, 2013), subcortical structures are thought to modulate the process of action selection, given afferent cortical signals (Koziol & Budding, 2009). Regarding the other identified regions, we can speculate that their pericallosal situation and their proximity to subcortical regions supports interhemispheric communication and the integration of cortico-subcortical loops (Koziol & Budding, 2009; van der Knaap & van der Ham, 2011). We provided in our analyses a numerical assessment of their consistency in the context of our model. Their functional relevance remains to be further validated in neurophysiological studies involving tailored experimental protocols, and the present work can guide future research investigating brain regions that underlie task-specific control.

### Distinguishing Resting-State From Task Conditions

In this study, we applied our approach to both resting-state and task-based FC without a priori distinction, although their properties are different (Deco et al., 2011) and resting-state was the only condition that did not require any active involvement of the individuals. Interestingly, our method captured the singularity of resting-state in several regards: The matching between structure-informed and empirical FC is lower (Figure 2A–D) and requires more input regions (Figure 2B–E). Moreover, the input set related to resting-state is distinct from that of task conditions (Figure 4C) and includes more regions belonging to the frontoparietal subsystem and to the default mode network (Figure 4A).

The larger variability of resting-state connectivity across individuals compared with task-based FC, as individuals are left to wander freely, can influence the significance of the group-level resting-state FC (see the Methodological Considerations section below). This could explain the lower correlation score obtained at the group level (Figure 2A). However, we also observed lower correlation scores at the individual level (Figure 2D) and the overlap of consensus input sets across individuals in resting-state is in the same range as other tasks (Figure 2F). This suggests that there exists a common set of control ROIs driving resting-state that is detected by our model. Further analyses of these regions at the individual level are required to validate their physiological role.

The gradient of structure-function coupling observed for the resting-state in Figure 3D, from high correlation in primary sensory areas to low correlation in regions associated with more abstract functions, is consistent with recent studies (Liégeois, Santos, Matta, Van De Ville, & Sayed, 2020; Preti & Van De Ville, 2019; Vázquez-Rodríguez et al., 2019). It has been suggested that the stronger correspondence between structure and function in visuomotor networks can support the fast reaction to peripheral inputs (Preti & Van De Ville, 2019), while the structure-function decoupling in transmodal regions can promote their involvement in higher order cognitive functions (Vázquez-Rodríguez et al., 2019). During active tasks, the overall structure-function coupling is stronger than in resting-state, indicating that the brain anatomy can support a variety of functional configurations that adjust to the ongoing task-demand. Note that we computed the correlation score for subsystems including connections linking different subsystems. Excluding these connections modifies the results reported in Figure 3D and the associated interpretation (see Supplementary Note 4).

Accumulating evidence from fMRI studies speculate that resting-state FC forms a “standard” architecture in which segregated functional subsystems are represented, and which supports the transfer of information related to the implementation of tasks (Cole et al., 2014; Deco et al., 2011; Greicius, Krasnow, Reiss, & Menon, 2003; Ito et al., 2017; van den Heuvel & Pol, 2010; Yeo et al., 2011). This could explain why, from a controllability viewpoint, our results distinguish rest (the passive, default state) from task conditions (the active, target states). Following the hypothesis that resting-state connectivity supports task implementation, an extension of this study consists in applying our framework to the graph structure defined by resting-state FC instead of the connectome, in order to investigate which brain regions drive the rest-to-task transitions.

### Methodological Considerations

Our study relies on several methodological choices. In the construction of the connectome matrix, we defined the weight of a structural connection between two regions as the streamline density between these regions, that is, the number of reconstructed streamlines normalized by the size of the ROIs they are linking (Hagmann et al., 2008). This normalization is used in order to mitigate the bias due to the variable size of ROIs (see Supplementary Note 2). Alternative weightings exist for structural connections, and which one is the most appropriate remains an open question (Oldham et al., 2020). In order to derive the group-level FC matrices, we computed the entry-wise average of individual-level matrices. Other approaches include computing the pairwise correlations of the concatenation of individual-level BOLD time series (Liégeois et al., 2020) or computing the barycenter of individual-level matrices, using the fact that they belong to the manifold of positive semi-definite matrices (Venkatesh, Jaja, & Pessoa, 2020). Finally, our approach assumes that FC is defined as the linear correlation between activity time series. More complex measures of FC, such as partial correlations (Liégeois et al., 2020) or mutual information (Hlinka, Paluš, Vejmelka, Mantini, & Corbetta, 2011), could provide complementary insights into the structure-function relationship but would require adjustments in the derivation of structure-informed FC.

### Conclusion and Future Work

This report presented a system-theoretic framework for identifying potential state-specific control regions through a model linking structure and function in human brain networks. In this respect, it linked concepts of brain communication dynamics and connectome controllability. We expect that future research, for instance in clinical populations, will further validate the proposed approach by studying the impact of neurological deficits and lesions on the identified control regions. This work could in turn guide physiological studies investigating the role of particular regions in controlling brain processes. Future work should also analyze individual differences in the identified control regions and their possible relation to behavior.

## MATERIALS AND METHODS

### Dataset

We retrieved the preprocessed “100 unrelated subjects” dataset of the Human Connectome Project (HCP) database (https://db.humanconnectome.org/), HCP 1200 release (Van Essen et al., 2013). All individuals (54 females, 46 males, 22–36 y.o.) gave written informed consent to the HCP consortium. Scanning protocols were approved by the local Institutional Review Board at Washington University in Saint Louis. Acquisition parameters are detailed in previous HCP reports (Glasser et al., 2013; Van Essen et al., 2013; Van Essen et al., 2012). Preprocessing consisted of HCP minimal preprocessing pipelines (Glasser et al., 2013). We applied further processing steps in agreement with previously published studies using HCP data (Amico et al., 2019; Rosenthal et al., 2018; Tipnis et al., 2018).

#### Parcellation.

We used the cortical parcellation introduced by Destrieux and colleagues (Destrieux et al., 2010) and composed of 148 nonoverlapping regions of interest (ROIs). Subcortical structures (thalamus, caudate nucleus, putamen, pallidum, hippocampus, amygdala, accumbens nucleus) and cerebellum were extracted using the FMRIB Software Library (Jenkinson, Beckmann, Behrens, Woolrich, & Smith, 2012) and added to the parcellation for completeness, bringing the number of ROIs to *n* = 164.

#### Connectome reconstruction.

The processing of diffusion data was conducted for each individual using state-of-the-art methods implemented in the MRtrix3 toolbox (Tournier et al., 2019). In summary, a tissue-segmented image was generated (MRtrix command 5ttgen) in order to perform anatomically constrained tractography (Smith, Tournier, Calamante, & Connelly, 2012). Then, multi-shell, multi-tissue response functions were computed (MRtrix command dwi2response msmt_5tt) in order to inform the constrained spherical deconvolution (MRtrix command dwi2fod msmt_csd; Jeurissen, Tournier, Dhollander, Connelly, & Sijbers, 2014). Probabilistic tractography (MRtrix command tckgen) was performed using a second-order integration over fiber orientation distributions (iFOD2 method; Tournier, Calamante, & Connelly, 2010) to allow for a more precise fiber tracking through crossing regions. This produced an initial tractogram composed of 10 million streamlines. The tractogram was corrected (SIFT2 approach, MRtrix command tcksift2) by assigning a weight to each streamline such that the weighted contribution of all streamlines to the spherical deconvolution diffusion model matches as well as possible the fiber orientation distribution lobe integrals of the diffusion data (Smith, Tournier, Calamante, & Connelly, 2015). This post hoc operation produced a more biologically meaningful representation of white matter tracts. Eventually, we built the adjacency matrix **S** of the connectome by computing the fiber density between each pair of previously defined ROIs (MRtrix command tck2connectome with option –scale_invnodevol). The group-average adjacency matrix is obtained as the entrywise average of the *K* = 100 individual-level adjacency matrices. Both group-average and individual matrices were kept unthresholded. Tractograms composed of 1 million or 100,000 streamlines were shown to produce a group-level matrix that is highly correlated (*r* > 0.99) with that used in the main analysis (see Supplementary Figure S12).

#### Empirical functional connectivity.

We included fMRI data acquired during resting-state and seven tasks. The emotional processing task (EMOTION) consisted of recognizing which of the two faces (resp. shapes) presented at the bottom of a screen matched the one presented at the top of the screen. Faces were aimed to represent either anger or fear. In the gambling task (GAMBLING), participants played a card guessing game in order to win or lose money. The acquisition comprised neutral blocks, blocks with mostly reward trials, and blocks with mostly loss trials. The language processing task (LANGUAGE) alternated story blocks and math blocks. In story blocks, participants had to answer a two-choice question after the hearing of a brief story. In math blocks, participants had to choose the right answer out of two after hearing an arithmetic operation. In the motor task (MOTOR), participants had to move either their fingers (left or right), their toes (left or right), or their tongue following visual cues on a screen. In the relational processing task (RELATIONAL), participants were presented with pairs of objects on a screen. Each object was one shape filled with one texture. Participants were asked to determine what dimension (shape or texture) differs between the objects. The social cognition task (SOCIAL) consisted of video clips presenting objects (squares, circles, triangles) either interacting in some way or moving randomly. Participants were asked to decide whether objects had an interaction or not, or not sure. In the working-memory task (WM), participants were presented with pictures to be memorized (zero-back and two-back trials). Separate blocks presented pictures of places, tools, faces, and body parts. Full details about the fMRI task protocols along with the references from which they are derived are available in the HCP 1200 reference manual.

The preprocessing of fMRI data included distortion correction, subject motion correction, intensity normalization, and registration to standard MNI space (Glasser et al., 2013). Resting-state blood-oxygenation-level dependent (BOLD) time series were filtered in forward and reverse directions (first-order Butterworth, bandpass = [0.001, 0.08] Hz; see Power et al., 2014). We did not regress out the global signal in the main analysis; the impact of global signal regression is discussed in Supplementary Note 3. For both resting-state and task fMRI, the voxel time series were then z-scored and averaged in each ROI using the Connectome Workbench toolbox (Marcus et al., 2011) and excluding outlier time points outside 3 standard deviations from the mean (Workbench command -cifti-parcellate). Empirical functional connectivity (FC) matrices **F**_*emp*_ were obtained by computing Pearson’s correlation coefficient between each pair of resulting time series. For each task, FC matrices of both fMRI phase encoding directions (left-to-right and right-to-left) were averaged in order to reduce the effect of artifactual noise. For resting-state, the four resulting matrices (two scans, two phase encoding directions) were averaged for the same reason. The group-average FC matrix (for each task and resting-state) is obtained as the entrywise average of the *K* = 100 individual-level FC matrices. Both group-average and individual matrices were kept unthresholded.

### State Correlation Matrix of a Linear Diffusion Process Driven by White Noise

We consider the following linear discrete time-invariant dynamics:

$$x\left(k+1\right)=Ax\left(k\right)+Bu\left(k\right).$$ (5)

We excite the system with white noise signals **u**, and we assume that **x** is centered, **A** is stable, the input signals are not correlated with the initial state of the system (i.e., 𝔼{**x**(0)**u**^*T*^(*k*)} = **0**, ∀*k*), and input signals in **u** have unit variance. We compute the steady-state covariance matrix **Σ** = Cov(**x**) of System 5 as follows:

$$\begin{array}{c} \Sigma \left(k+1\right)=𝔼\left\{x\left(k+1\right)x^{T}\left(k+1\right)\right\}\\ \;=𝔼\left\{\left(Ax\left(k\right)+Bu\left(k\right)\right){\left(Ax\left(k\right)+Bu\left(k\right)\right)}^{T}\right\}\\ \;=A𝔼\left\{x\left(k\right)x^{T}\left(k\right)\right\}A^{T}+A𝔼\left\{x\left(k\right)u^{T}\left(k\right)\right\}B^{T}\\ \;+B𝔼\left\{u\left(k\right)x^{T}\left(k\right)\right\}A^{T}+B𝔼\left\{u\left(k\right)u^{T}\left(k\right)\right\}B^{T}\\ \;=A\Sigma \left(k\right)A^{T}+{BB}^{T}\;\text{using}\;\text{the}\;\text{assumptions}\;\text{on}\;u\left(k\right)\\ \;\Rightarrow \Sigma ={A\Sigma A}^{T}+{BB}^{T}\;\text{in}\;\text{steady-state}. \end{array}$$

We notice that excitation signals with non-unit variance would result in a scaling of matrix **B**, which would not affect further results.

Defining **P** as the diagonal matrix containing only the diagonal entries of **Σ** (i.e., the states’ variances), we can apply a symmetric normalization to the steady-state covariance matrix to obtain a pairwise correlation matrix that we use as a model of functional connectivity:

$$\begin{array}{c} \tilde{\Sigma }=P^{-1/2}{\Sigma P}^{-1/2},\\ F_{SI}=\tilde{\Sigma }. \end{array}$$

In this study, we consider the case of a diffusion process unfolding on the connectome, as proposed by Abdelnour and colleagues (Abdelnour et al., 2014). The state transition matrix **A** has the following form:

$$A=e^{-\beta 𝓛}.$$

Here, with **D** being the diagonal matrix of the weighted degree of the ROIs, the matrix 𝓛 = **D**^−1/2^(**D** − **S**)**D**^−1/2^ is the normalized Laplacian of the connectome. The parameter *β* = $\overset{ˉ}{\beta }$Δ*T* accounts for the diffusion time constant $\overset{ˉ}{\beta }$ and the sampling time Δ*T* of the process. We chose to hold this parameter out of the optimization and to set $\overset{ˉ}{\beta }$ = 1 and Δ*T* = TR, where TR = 0.72 s is the repetition time of the fMRI data (Van Essen et al., 2013). This choice is arbitrary and the optimal *β* is state-dependent (see Supplementary Figure S14), although choosing *β* ∈ [0.1, 4.0] has a weak impact on the correlation score and produces similar input sets (see Supplementary Figure S15). Note that any hyperparameter coming with the chosen transition matrix can be included in the optimization, at the expense of a possibly prohibitive computational cost.

In order to ensure that System 5 unforced dynamics is intrinsically stable so that the activities decay to zero in the absence of control signals (see Gu et al. [2015], Kim et al. [2018], and the detailed discussion in Karrer et al. [2020]), the entries of matrix **A** were further divided by 1 + *λ*_max_(**A**), where *λ*_max_(**A**) is the largest eigenvalue of **A**. In our case, the smallest eigenvalue of 𝓛 is always 0 since the Laplacian of an unsigned graph is positive semi-definite, and always possesses a zero eigenvalue (Mohar, Alavi, Chartrand, & Oellermann, 1991). Therefore, *λ*_max_(*e*^−*β*𝓛^) is always equal to 1.

### Correlation Score

The similarity between **F**_*SI*_ and **F**_*emp*_ is computed as the entry-wise Pearson’s correlation coefficient *r*, following previous work (Abdelnour et al., 2014; Finn et al., 2015; Goñi et al., 2014; Mišić et al., 2015; Tipnis et al., 2018). Denoting $\overset{△}{F}$_*SI*_ (resp. $\overset{△}{F}$_*emp*_) as the vectorized version of the upper triangular part of **F**_*SI*_ (resp. **F**_*emp*_), we define *r* as

$$r=\text{corr}\left({\overset{△}{F}}_{SI},{\overset{△}{F}}_{emp}\right).$$ (6)

### Finding the Optimal Set of Control Regions: Genetic Algorithms

Given the combinatorial nature of the optimization Problem 4, we must resort to heuristic methods in order to approach optimal solutions, without guarantee of optimality. A convenient choice is the family of genetic algorithms (Wolsey & Nemhauser, 1999). Here, the steps involved in the genetic algorithm that we used are (a) generating a random population of admissible input sets, (b) selecting the best input sets in the population, (c) breeding a new generation of solutions by crossovers between selected input sets, (d) applying random modifications in the new population to avoid getting trapped in a local optimum, and (e) repeating the process until no more improvement is achieved after a given number of iterations. In the present study, we used the Matlab implementation of genetic algorithms, from the Global Optimization Toolbox, with default options and parameters. The Matlab code used to produce the results in this report is available online (https://github.com/bchiem42/Structure-informed-FC; Chiêm, 2020).

### Consensus Input Set

In order to mitigate the lack of optimality guarantee of genetic algorithms, we compute multiple solutions using random initializations. We define the consensus input set as the set of ROIs selected at least *k* times. Inspecting the histogram of ROI selections allows us to set the threshold value *k*. In our analysis, the distribution of ROI selections is bimodal (see Supplementary Figure S3 for a typical example). Therefore, choosing different thresholds *k* between both modes of the histogram has a limited impact on the results (see Supplementary Figure S4). Note that choosing a threshold too low could produce a consensus input set of which the cardinality is higher than the number of input nodes allowed in the optimization, especially when few input nodes are allowed.

### Baselines

In order to assess how well our approach maps structure to function, we provide three baseline values.

#### Baseline 1.

This is the Pearson’s correlation coefficient between the vectorized upper-triangular of the adjacency matrix of the connectome **S** and the empirical FC matrix **F**_*emp*_, without any transformation.

#### Baseline 2.

We randomly relabel the ROIs of the connectome matrix **S** while keeping **F**_*emp*_ unchanged, and then apply our method. This null model breaks the ROI-to-ROI correspondence between structure and function and preserves all network properties of the connectome. In particular, it preserves the distribution of weighted degrees and allows us to test whether the selection of control ROIs is exclusively driven by their degree. In the Results, we report the maximum correlation score obtained over 30 random relabeling.

#### Baseline 3.

In order to assess the usefulness of solving the optimization Problem 4 to identify optimal input sets, we compute the correlation score between **F**_*emp*_ and **F**_*SI*_ obtained with an input set drawn uniformly at random, with cardinality *m* ∈ [37, 43], following the result depicted in Figure 2B. In the Results, we report the maximum correlation score obtained over 30 random input sets.

### Modal Controllability

Given a system defined on a network of *n* nodes, modal controllability is a nodal property that quantifies the ability of a single node to steer the system towards states requiring substantial input energy (Gu et al., 2015; Karrer et al., 2020; Pasqualetti et al., 2014). We compute the modal controllability *ϕ*_*i*_ of node *i* from the eigenvalues *λ* and the eigenvectors **v** of the adjacency matrix **S** of the connectome:

$$\phi_{i}=\sum_{j=1}^{n}\left(1-\lambda_{j}^{2}\left(S\right)\right)v_{ij}^{2}.$$

In this work, we computed modal controllability using the Matlab implementation provided by the authors of Gu et al. (2015).

### 2D Visualization of Input Sets Using *t*-Distributed Stochastic Neighbor Embedding

In a network of *n* nodes, we represent an input set as an *n*-dimensional binary vector indicating which node is selected (1) or not (0). In order to visualize how multiple input sets relate to each other, we can use dimensionality reduction to embed the *n*-dimensional vectors in two dimensions. In particular, the *t*-distributed stochastic neighbor embedding (van der Maaten & Hinton, 2008) aims at finding a low-dimensional representation of high-dimensional vectors while preserving their local structure, such that similar vectors are represented by close points in 2D and vice versa, with high probability. In this work, we used the Jaccard index to measure the similarity between vectors.

### Jaccard Index

The Jaccard index *J* between two sets 𝒮_1_ and 𝒮_2_ measures the overlap between these sets and is computed as

$$J\left({𝒮}_{1},{𝒮}_{2}\right)=\frac{\left|{𝒮}_{1}\cap {𝒮}_{2}\right|}{\left|{𝒮}_{1}\cap {𝒮}_{2}\right|},$$ (7)

with *J* = 0 indicating no overlap and *J* = 1 indicating perfect overlap. To derive the expected value of *J*, we consider a set of *n* elements from which we draw two subsets 𝒮_1_ and 𝒮_2_ having the same cardinality *m* and whose elements are chosen uniformly at random. We denote the number of common elements between 𝒮_1_ and 𝒮_2_ as |𝒮_1_ ∩ 𝒮_2_| = *k*. The corresponding Jaccard index is

$$J\left({𝒮}_{1},{𝒮}_{2}\right)=\frac{k}{2m-k}.$$

Now, the probability that the number of common elements is exactly *k* is

$$P\left(\left|{𝒮}_{1}\cap {𝒮}_{2}\right|=k\right)=\frac{\left(\begin{array}{c} m\\ k \end{array}\right)\left(\begin{array}{c} n-m\\ m-k \end{array}\right)}{\left(\begin{array}{c} n\\ m \end{array}\right)},$$

since we have $\left(\begin{array}{c} n\\ m \end{array}\right)$ choices for the elements of 𝒮_1_ and $\left(\begin{array}{c} m\\ k \end{array}\right)\left(\begin{array}{c} n-m\\ m-k \end{array}\right)$ choices left for the elements of 𝒮_2_. Therefore, the expected value of the Jaccard index between two random sets of size *m* drawn from *n* elements is

$$𝔼\left\{J\left({𝒮}_{1},{𝒮}_{2}\right)\right\}=\sum_{k=0}^{m}\frac{\left(\begin{array}{c} m\\ k \end{array}\right)\left(\begin{array}{c} n-m\\ m-k \end{array}\right)}{\left(\begin{array}{c} n\\ m \end{array}\right)}\frac{k}{2m-k}.$$

For *n* = 164 and *m* = 40 (see Figure 2E), we obtain 𝔼{*J*} ≈ 0.1389.

## ACKNOWLEDGMENTS

The authors would like to thank Laurence Dricot for her helpful comments and suggestions. Benjamin Chiêm is a FRIA (F.R.S.-FNRS) fellow. Data were provided by the Human Connectome Project, WU-Minn Consortium (principal investigators: David Van Essen and Kamil Ugurbil; 1U54MH091657) funded by the 16 NIH Institutes and Centers that support the NIH Blueprint for Neuroscience Research; and by the McDonnell Center for Systems Neuroscience at Washington University.

## SUPPORTING INFORMATION

Supporting information for this article is available at https://doi.org/10.1162/netn_a_00192.

## AUTHOR CONTRIBUTIONS

Benjamin Chiêm: Conceptualization; Data curation; Formal analysis; Methodology; Validation; Visualization; Writing – original draft; Writing – review & editing. Frédéric Crevecoeur: Conceptualization; Methodology; Supervision; Validation; Writing – review & editing. Jean-Charles Delvenne: Conceptualization; Methodology; Supervision; Validation; Writing – review & editing.

## FUNDING INFORMATION

Benjamin Chiêm, Fonds pour la Formation à la Recherche dans l'Industrie et dans l’Agriculture - FRIA (F.R.S.-FNRS) (BE), Award ID: 1.E051.18+F. Frédéric Crevecoeur, Fonds de la Recherche Scientifique (F.R.S.-FNRS) (BE), Award ID: 1.C.033.18F.

## TECHNICAL TERMS

Controllability: The ability of a system to move from any initial state to any final state, given external control signals.
Gramian: In control theory, a fundamental matrix providing quantitative information about how “difficult” it is to control a linear system to a given state.
Structure-informed functional connectivity: A model of functional coactivations developing on the brain’s anatomical structure and driven by a set of control regions.
Genetic algorithm: A heuristic optimization method that iteratively combines multiple candidate solutions in order to form a near optimal one.
Consensus input set: The set of control regions frequently selected by repeated runs of a randomized method.
Jaccard index: A measure of the overlap of the elements of two sets.
Subsystem: A subset of brain areas associated with a particular function.
*t*-SNE: A nonlinear dimensionality reduction method allowing the visualization of high-dimensional data points in 2D while preserving their neighborhood structure.
Modal controllability: A measure of the ability of a single node to drive a network towards difficult-to-reach states.

## External Supplement

https://github.com/bchiem42/Structure-informed-FC
