# Supplementary material for: Structure-informed functional connectivity driven by identifiable and state-specific control regions
Source: Netw Neurosci. 2021 Jun 21;5(2):591–613. doi: 10.1162/netn_a_00192 (PMC8233121; doi:10.1162/netn_a_00192)
Supplement: Supplementary file 1 [file netn-05-591-s001.pdf]

Chiêm, B., Crevecoeur, F. & Delvenne, J. (2021). Supporting information for "Structure-Informed Functional Connectivity Driven by Identifiable and State-Specific Control Regions." *Network Neuroscience*. Advance publication. https://doi.org/10.1162/netn_a_00192

Structure-informed functional connectivity driven by identifiable and state-specific control regions
# - Supplementary Material -

Benjamin Chiêm[1,2], Frédéric Crevecoeur[1,2], and Jean-Charles Delvenne[1]

[1]*Institute of Communication Technologies, Electronics and Applied Mathematics, Department of Mathematical Engineering, Université catholique de Louvain, Louvain-la-Neuve, Belgium*
[2]*Institute of Neuroscience, Division of Systems and Cognitive Neuroscience, Université catholique de Louvain, Brussels, Belgium*

Supplementary Notes

Note 1 : Linear models to map structure and function in brain networks

We use a linear time-invariant model of the form

$$\mathbf{x}(k+1) = \mathbf{A}\mathbf{x}(k) + \mathbf{B}\mathbf{u}(k) \tag{1}$$

where $\mathbf{x}(k)$ describes the level of neurophysiological activity at time k in each node of a brain network. The model parameters are the system matrix $\mathbf{A}$, the input matrix $\mathbf{B}$ and the input signals $\mathbf{u}$.

Roberto Galán proposed such a model based on the linearization and discretization of a general Wilson-Cowan model [1]. The author obtained $\mathbf{A}$ as a linear transformation of the structural connectivity matrix (i.e. the connectome), fixed $\mathbf{B}$ as the identity matrix, modelled $\mathbf{u}$ as white noise signals and derived an analytical expression of the state-covariance matrix of the nodal dynamics. This model was further explored by Honey and colleagues [2]. Based on this work, Gu et al. leveraged the established theory of linear systems to investigate the controllability of brain networks [3]. By choosing $\mathbf{B}$ as the i-th canonical vector, they derived quantitative control properties of each node i through the computation of the controllability Gramian. Later, Gu et al. focused on optimal trajectories of brain state transitions [4] and computed the input signals $\mathbf{u}$ minimizing the energy for the transitions between predefined brain states. For that, they took a hypothesis-driven approach and fixed the input matrix $\mathbf{B}$ based on *a priori* knowledge. The authors pointed out to the theoretical and empirical motivations of considering a *set* of control regions (*multipoint control*) instead of a single input node. However, the question of how to identify the control set associated with a given brain state from empirical data remains challenging.

In parallel, recent studies investigated how information propagates in the white matter wiring in order to generate the observed patterns of functional activity [5]. A spectrum of communication strategies have been investigated [6] among which several models based on information diffusion [7, 8, 9, 10]. These models account for the autonomous dynamics of System 1 through modifications of the system matrix $\mathbf{A}$ [11] in the absence of external stimulation ($\mathbf{B} = \mathbf{0}$).

Here, we propose a principled method to identify state-specific sets of control regions from empirical data. For that, we use System 1 in which $\mathbf{A}$ describes a Laplacian diffusion dynamics [7] although the general framework is valid for other dynamics. We derive a model of correlation-based functional connectivity that is linked to the controllability Gramian and find the input matrix $\mathbf{B}$ such that the similarity between modelled and empirical functional connectivity is maximized, assuming white driving noise signals $\mathbf{u}$.

Note 2 : Structural connectivity weighting

In our analysis, we weighted the structural connection between two regions of the connectome as the number of streamlines reconstructed by the tractography algorithm between both regions, normalized by the volume of the regions. This process has been proposed by Hagmann and colleagues [12] in order to mitigate the bias due to the variable size of ROIs, larger ROIs receiving more reconstructed streamlines. In Supplementary Figure S2, we observe that some regions such as the cerebellum are particularly affected by this normalization. Alternative weightings of structural connections exist in the literature, related to the length of reconstructed streamlines, their fractional anisotropy or the apparent diffusion coefficient for instance. Choosing the edges weighting scheme remains an open question in diffusion MRI connectomics [13] and our results should be interpreted with respect to this choice as the nodal strength has been shown to be linked to the chance of a ROI to be selected as an input node.

Note 3 : Impact of Global Signal Regression

Global Signal Regression (GSR) is a highly debated preprocessing operation [14, 15, 16, 17] because the global signal is thought to include components of both neuronal and non-neuronal origin. Here, we can anticipate that GSR will have a negative impact on the correlation score between structure-informed and empirical functional connectivity. On the one hand, the solution $\mathbf{\Sigma}$ of the Lyapunov equation used in our our model is written

$$\mathbf{\Sigma} = \sum_{i=0}^{\infty} \mathbf{A}^i \mathbf{B} \mathbf{B}^T (\mathbf{A}^T)^i \tag{2}$$

and cannot have negative entries with our choice for $\mathbf{A}$ and $\mathbf{B}$. On the other hand, GSR has been shown to introduce negative correlations in empirical FC [18]. Therefore, our model is expected to show lower performance when GSR is applied to the fMRI data, because it cannot predict the negative correlations that are introduced. To illustrate this, we reproduced Figures 2A, 2B, 3A and 3B of the manuscript using fMRI data that underwent GSR. In Supplementary Figure S13A, we observe that correlation scores are indeed lower for all states, with the relational processing task being the most affected. Compared to the results of the main text, less control ROIs are selected for all states except the resting-state (Supplementary Figure S13B). Our previous observation that subcortical areas are consistently selected across states remains valid (Supplementary Figure S13C and D for the motor task and the resting-state respectively ; result not shown for the other tasks).

Note 4 : Structure-function correlation in open and closed subsystems

In order to compute the structure-function correlation score for a subsystem, one can consider two options : (i) comparing the edges with both extremities belonging to the subsystem ('closed' subsystem) or (ii) comparing the edges with *at least one* extremity belonging to the subsystem ('open' subsystem). The first option ignores all edges linking two subsystems while the second includes these edges in the computation of the correlation score for both subsystems. In the manuscript, Figure 3D is obtained with open subsystems and shows a gradient of structure-function coupling in resting-state, from high correlation in primary sensory areas to low correlation in regions associated with higher-order cognitive functions [19]. In Supplementary Figure S8, we show the result of the same analysis with closed subsystems. This result is similar to that reported by Tipnis et al. [20], and the gradient observed in resting-state is different from that of the manuscript. We also observe that the visual subsystem is the most affected by taking into account the edges linking different subsystems, indicating that the connections between visual areas and other systems show a closer structure-function relationship, compared to intrinsic connections of the visual subsystems.

References

[1] Roberto F Galán. On how network architecture determines the dominant patterns of spontaneous neural activity. *PloS one*, 3(5):e2148, 2008.

[2] Christopher J Honey, Olaf Sporns, Leila Cammoun, Xavier Gigandet, Jean-Philippe Thiran, Reto Meuli, and Patric Hagmann. Predicting human resting-state functional connectivity from structural connectivity. *Proceedings of the National Academy of Sciences*, 106(6):2035–2040, 2009.

[3] Shi Gu, Fabio Pasqualetti, Matthew Cieslak, Qawi K Telesford, B Yu Alfred, Ari E Kahn, John D Medaglia, Jean M Vettel, Michael B Miller, Scott T Grafton, et al. Controllability of structural brain networks. *Nature communications*, 6(1):1–10, 2015.

[4] Shi Gu, Richard F Betzel, Marcelo G Mattar, Matthew Cieslak, Philip R Delio, Scott T Grafton, Fabio Pasqualetti, and Danielle S Bassett. Optimal trajectories of brain state transitions. *Neuroimage*, 148:305–317, 2017.

[5] Andrea Avena-Koenigsberger, Bratislav Misic, and Olaf Sporns. Communication dynamics in complex brain networks. *Nature Reviews Neuroscience*, 19(1):17, 2018.

[6] Andrea Avena-Koenigsberger, Xiaoran Yan, Artemy Kolchinsky, Martijn van den Heuvel, Patric Hagmann, and Olaf Sporns. A spectrum of routing strategies for brain networks. *PLoS computational biology*, 15(3):e1006833, 2019.

[7] Farras Abdelnour, Henning U Voss, and Ashish Raj. Network diffusion accurately models the relationship between structural and functional brain connectivity networks. *Neuroimage*, 90:335–347, 2014.

[8] Joaquín Goñi, Martijn P Van Den Heuvel, Andrea Avena-Koenigsberger, Nieves Velez De Mendizabal, Richard F Betzel, Alessandra Griffa, Patric Hagmann, Bernat Corominas-Murtra, Jean-Philippe Thiran, and Olaf Sporns. Resting-brain functional connectivity predicted by analytic measures of network communication. *Proceedings of the National Academy of Sciences*, 111(2):833–838, 2014.

[9] Bratislav Mišić, Richard F Betzel, Azadeh Nematzadeh, Joaquin Goni, Alessandra Griffa, Patric Hagmann, Alessandro Flammini, Yong-Yeol Ahn, and Olaf Sporns. Cooperative and competitive spreading dynamics on the human connectome. *Neuron*, 86(6):1518–1529, 2015.

[10] Ruggero G Bettinardi, Gustavo Deco, Vasileios M Karlaftis, Tim J Van Hartevelt, Henrique M Fernandes, Zoe Kourtzi, Morten L Kringelbach, and Gorka Zamora-López. How structure sculpts function: unveiling the contribution of anatomical connectivity to the brain's spontaneous correlation structure. *Chaos: An Interdisciplinary Journal of Nonlinear Science*, 27(4):047409, 2017.

[11] Renaud Lambiotte, Roberta Sinatra, J-C Delvenne, Tim S Evans, Mauricio Barahona, and Vito Latora. Flow graphs: Interweaving dynamics and structure. *Physical Review E*, 84(1):017102, 2011.

[12] Patric Hagmann, Leila Cammoun, Xavier Gigandet, Reto Meuli, Christopher J Honey, Van J Wedeen, and Olaf Sporns. Mapping the structural core of human cerebral cortex. *PLoS Biol*, 6(7):e159, 2008.

[13] Stuart Oldham, Aurina Arnatkeviciute, Robert E Smith, Jeggan Tiego, Mark A Bellgrove, and Alex Fornito. The efficacy of different preprocessing steps in reducing motion-related confounds in diffusion mri connectomics. *NeuroImage*, 222:117252, 2020.

[14] Kevin Murphy and Michael D Fox. Towards a consensus regarding global signal regression for resting state functional connectivity mri. *Neuroimage*, 154:169–173, 2017.

[15] Jonathan D Power, Mark Plitt, Timothy O Laumann, and Alex Martin. Sources and implications of whole-brain fmri signals in humans. *Neuroimage*, 146:609–625, 2017.

[16] Matthew F Glasser, Timothy S Coalson, Janine D Bijsterbosch, Samuel J Harrison, Michael P Harms, Alan Anticevic, David C Van Essen, and Stephen M Smith. Using temporal ica to selectively remove global noise while preserving global signal in functional mri data. *NeuroImage*, 181:692–717, 2018.

[17] Kevin M Aquino, Ben D Fulcher, Linden Parkes, Kristina Sabaroedin, and Alex Fornito. Identifying and removing widespread signal deflections from fmri data: Rethinking the global signal regression problem. *NeuroImage*, 212:116614, 2020.

[18] Kevin Murphy, Rasmus M Birn, Daniel A Handwerker, Tyler B Jones, and Peter A Bandettini. The impact of global signal regression on resting state correlations: are anti-correlated networks introduced? *Neuroimage*, 44(3):893–905, 2009.

[19] Bertha Vázquez-Rodríguez, Laura E Suárez, Ross D Markello, Golia Shafiei, Casey Paquola, Patric Hagmann, Martijn P Van Den Heuvel, Boris C Bernhardt, R Nathan Spreng, and Bratislav Misic. Gradients of structure–function tethering across neocortex. *Proceedings of the National Academy of Sciences*, 116(42):21219–21227, 2019.

[20] Uttara Tipnis, Enrico Amico, Mario Ventresca, and Joaquin Goni. Modeling communication processes in the human connectome through cooperative learning. *IEEE Transactions on Network Science and Engineering*, 7(1):476–488, 2018.

Supplementary Figures

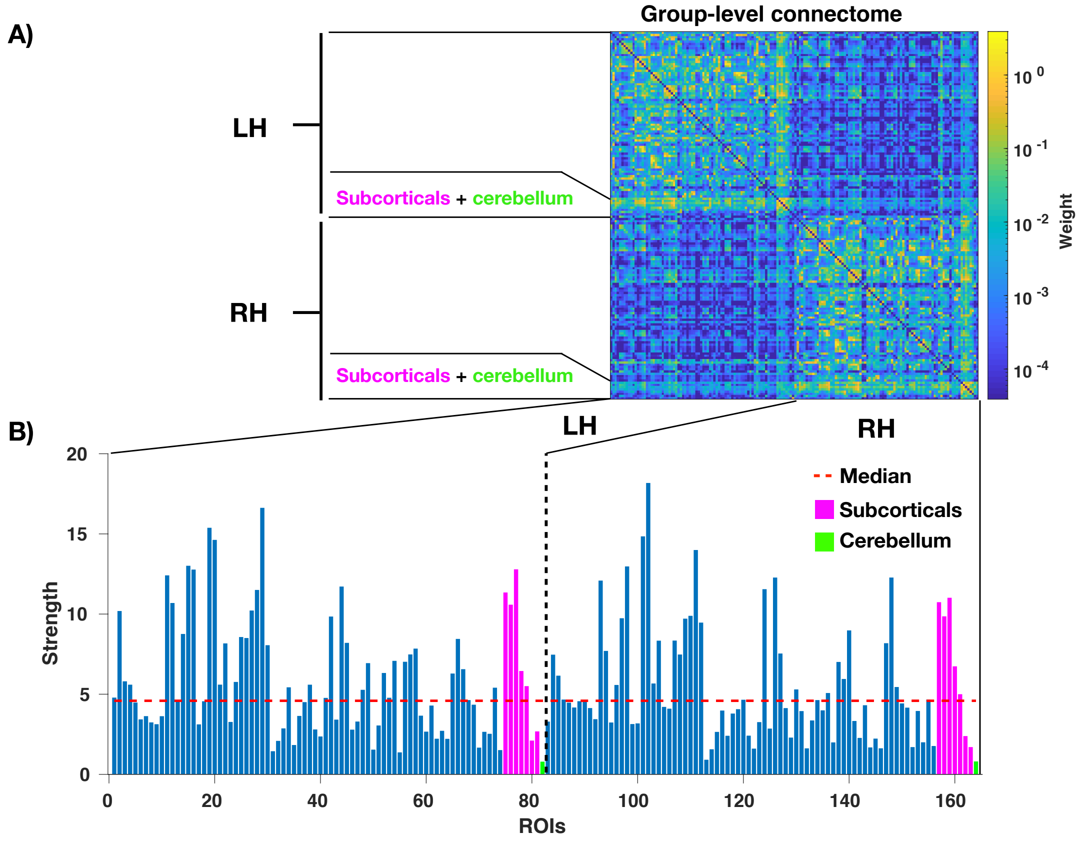

Figure S1: **Group-level connectome. A)** Adjacency matrix (Destrieux parcellation, 164 ROIs including subcorticals and cerebellum, streamline density weighting). LH: Left hemisphere. RH: Right hemisphere. **B)** Bar plot of the corresponding nodal strengths.

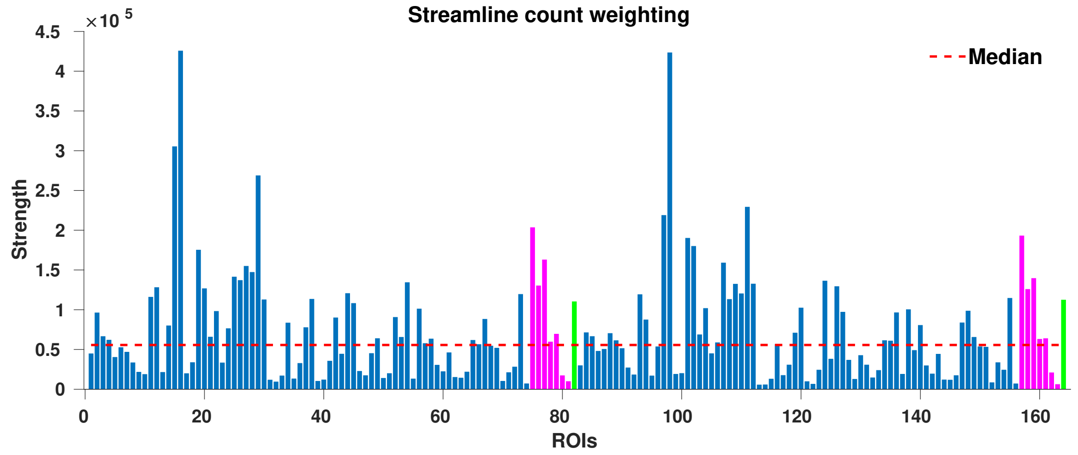

Figure S2: Bar plot of the nodal strengths in the connectome reconstructed using the streamline count weighting. Blue: cortical ROIS. Purple: subcorticals. Green: cerebellum.

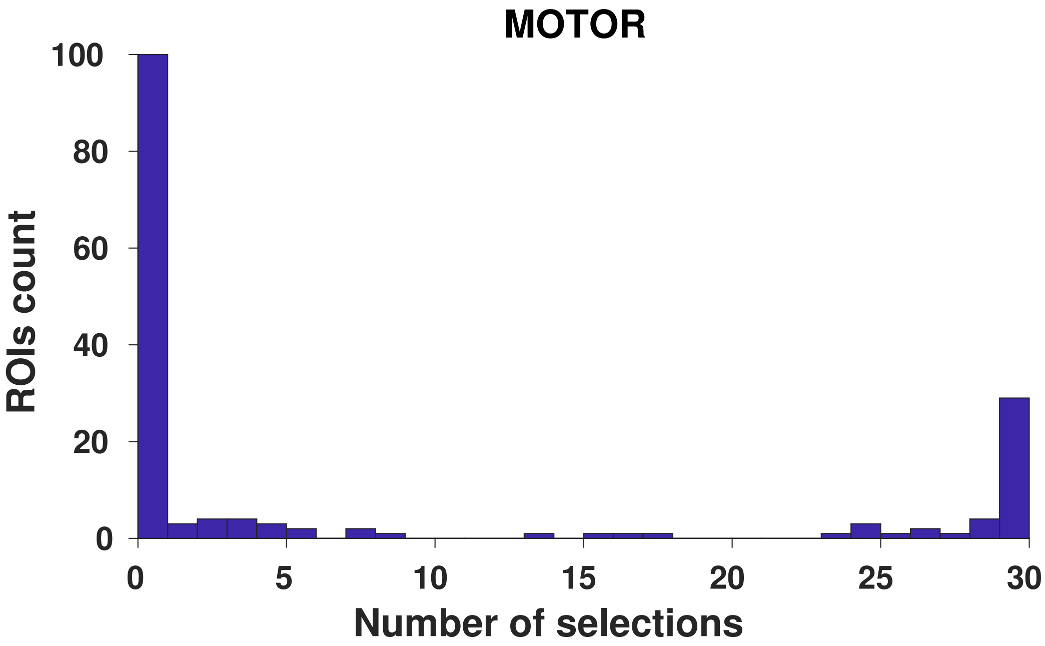

Figure S3: Histogram of ROIs selections (over 30 optimization runs) for the motor task, with no constraint on the number of input nodes allowed ($U = N$).

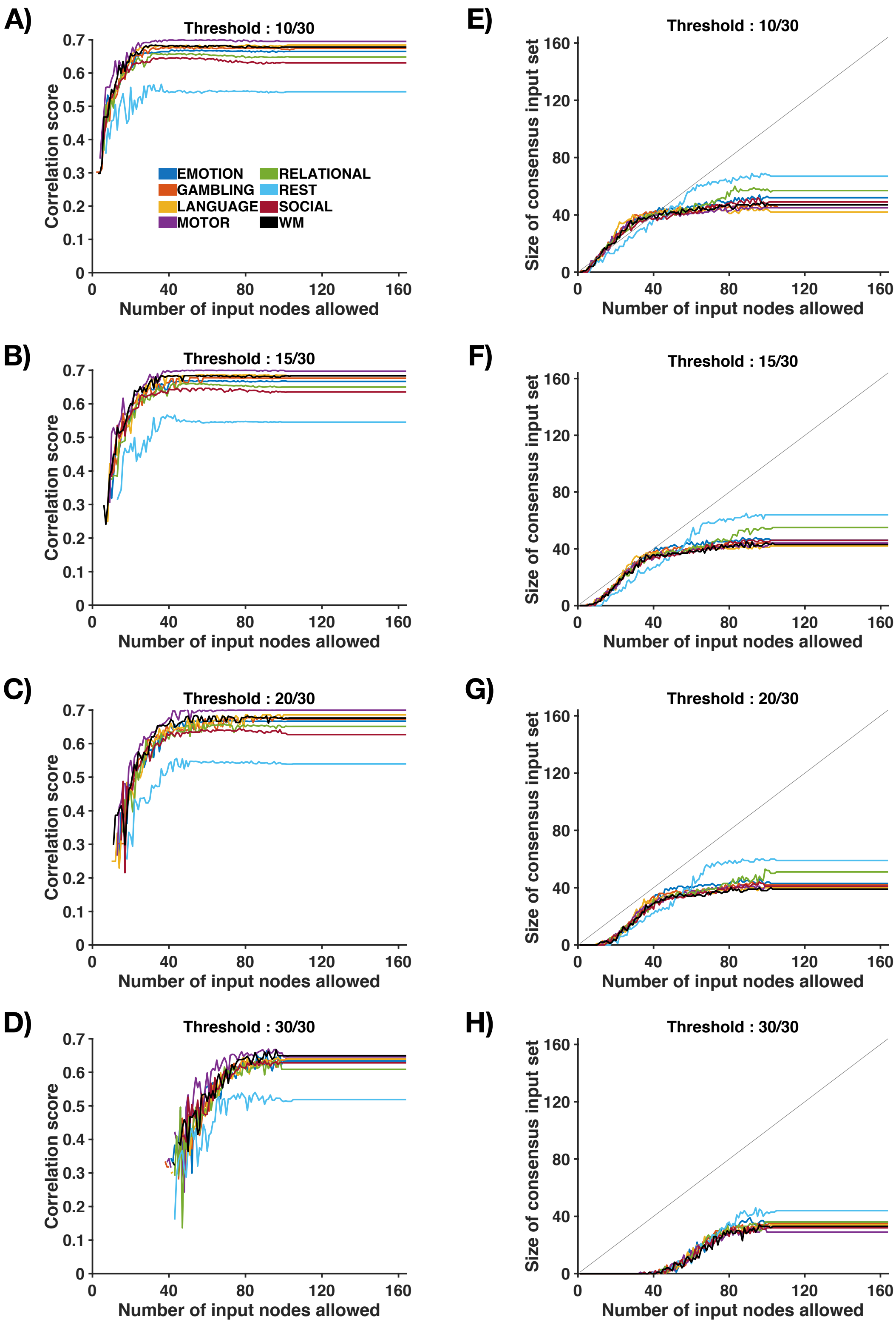

Figure S4: **Robustness with respect to consensus input set thresholds. A)** (resp. **B**, **C**, **D**) Correlation score between structure-informed and empirical functional connectivity with respect to the number of input nodes allowed U (group-level). $\mathbf{F}_{SI}$ is obtained using the consensus input set formed by ROIs selected at least 10 (resp. 15, 20, 30) times over 30 optimization runs. **E)** (resp. **F**, **G**, **H**) Size of the corresponding consensus input set with respect to the number of input nodes allowed U (group-level). The grey line denotes the identity function $y = x$.

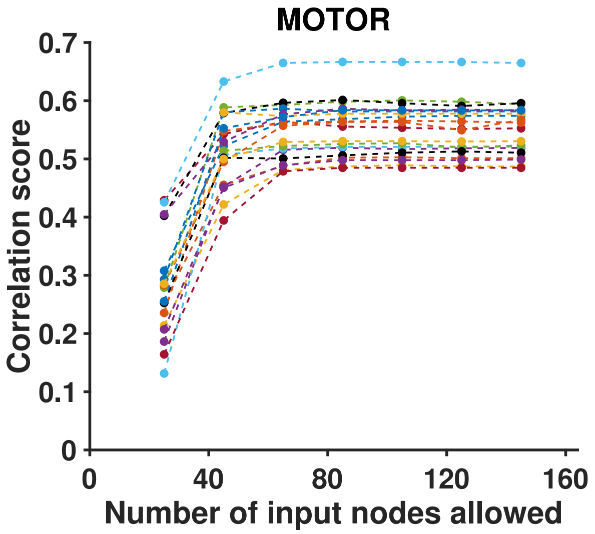

Figure S5: Correlation score between structure-informed and empirical functional connectivity with respect to the number of input nodes allowed $U \in \{5, 25, 45, 65, 85, 105, 125, 145\}$, for 20 randomly selected individuals. Left: motor task. Right: resting-state. The consensus input set is defined as the set of ROIs selected at least 25 times over 30 optimization runs.

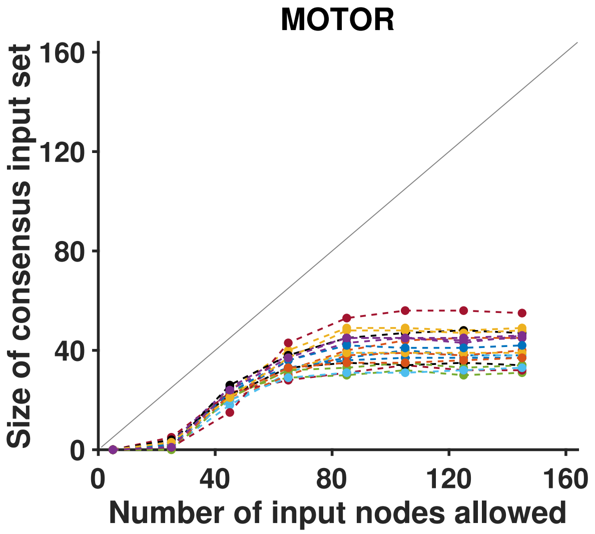

Figure S6: Size of the consensus input set with respect to the number of input nodes allowed $U \in \{5, 25, 45, 65, 85, 105, 125, 145\}$, for 20 randomly selected individuals. Left: motor task. Right: resting-state. The consensus input set is defined as the set of ROIs selected at least 25 times over 30 optimization runs.

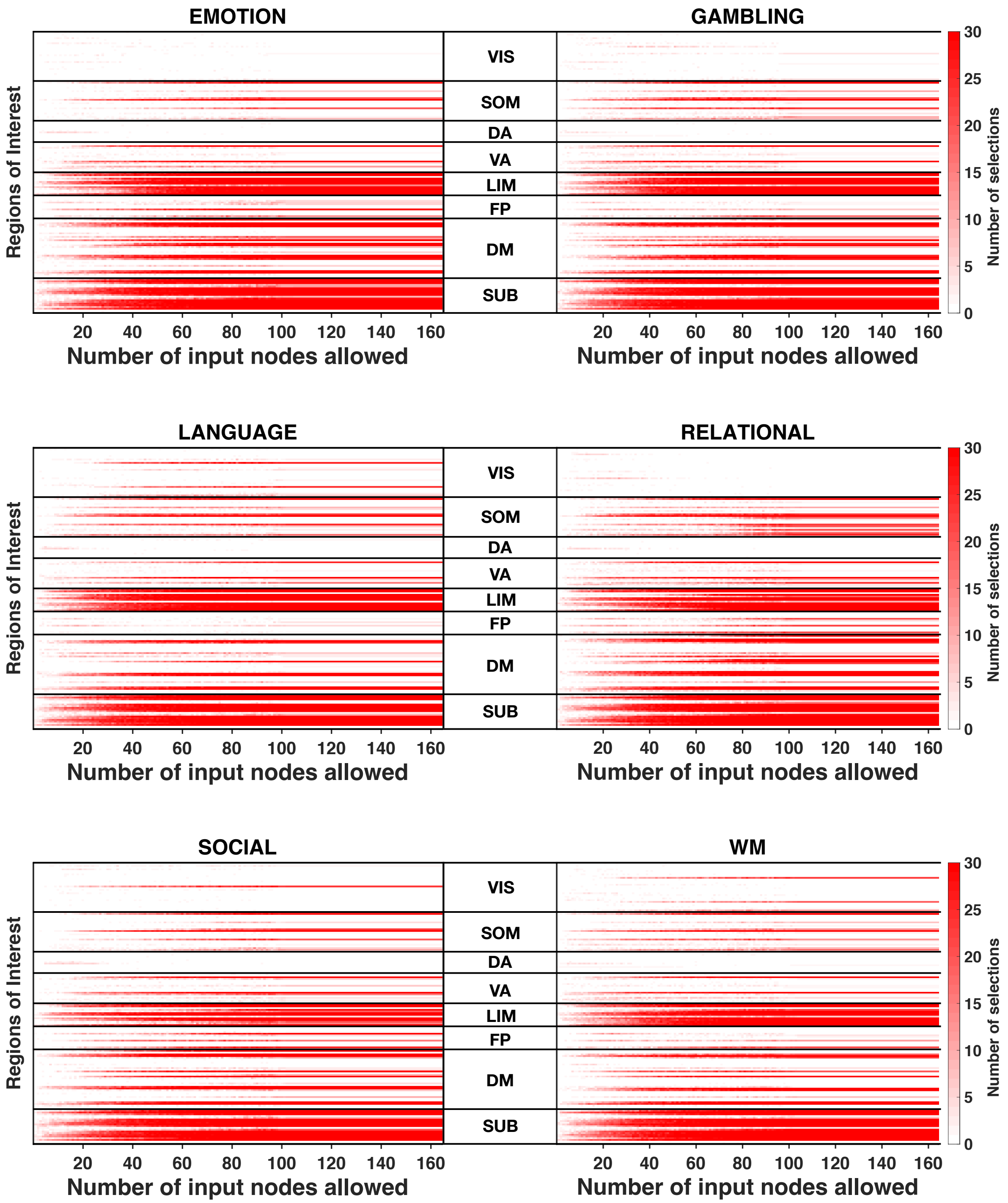

Figure S7: Analysis across functional subsystems (group-level). Evolution of the number of selections (from 0-white, to 30-red) of each Region of Interest (ROI) with respect to the number of input nodes allowed U for different tasks (MOTOR and REST are presented in the main manuscript). ROIs are arranged according to the functional subsystems described by Yeo and colleagues. The cerebellum is included in the "subcorticals" subsystem for visualisation and corresponds to the last two lines (left and right hemispheres)

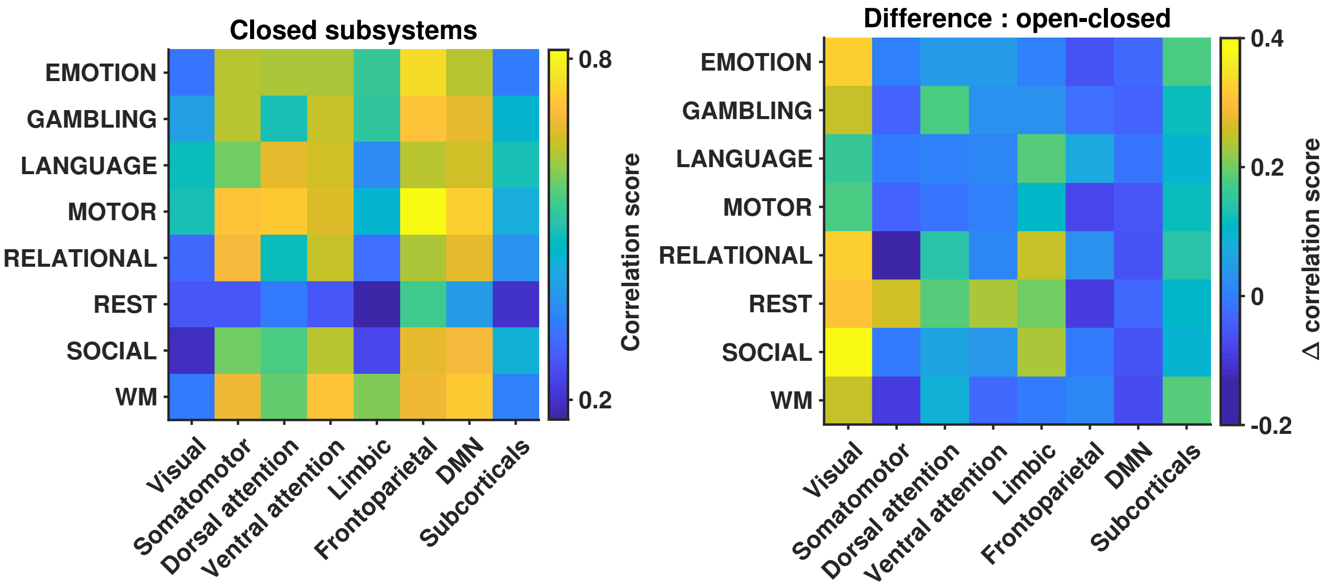

Figure S8: Left : Correlation between structure-informed and empirical functional connectivity with $U = N$, considering closed subsystems. Right : Difference of correlation scores across subsystems, when considering open (Figure 3D of the manuscript) or closed subsystems.

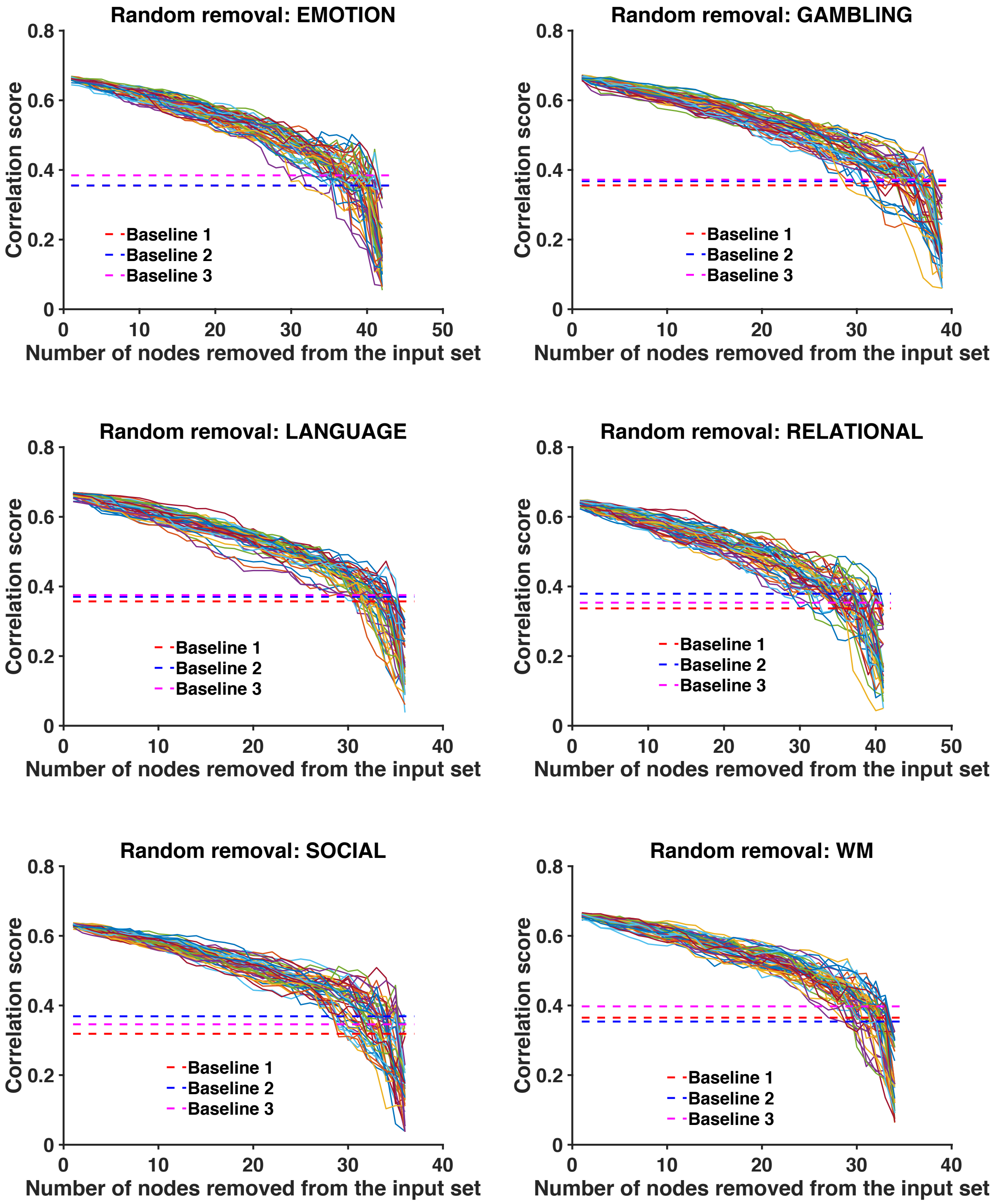

Figure S9: Robustness analysis. Evolution of the correlation between structure-informed $\mathbf{F}_{SI}$ and empirical functional connectivity $\mathbf{F}_{emp}$ as a function of the number of ROIs removed from the consensus input set. Dashed lines represent the three baselines, i.e. the correlation between $\mathbf{F}_{emp}$ and (i) the adjacency matrix of the connectome, (ii) $\mathbf{F}_{SI}$ based on a re-labelled connectome and (iii) $\mathbf{F}_{SI}$ obtained with a random input set. We consider 50 random removal orderings.

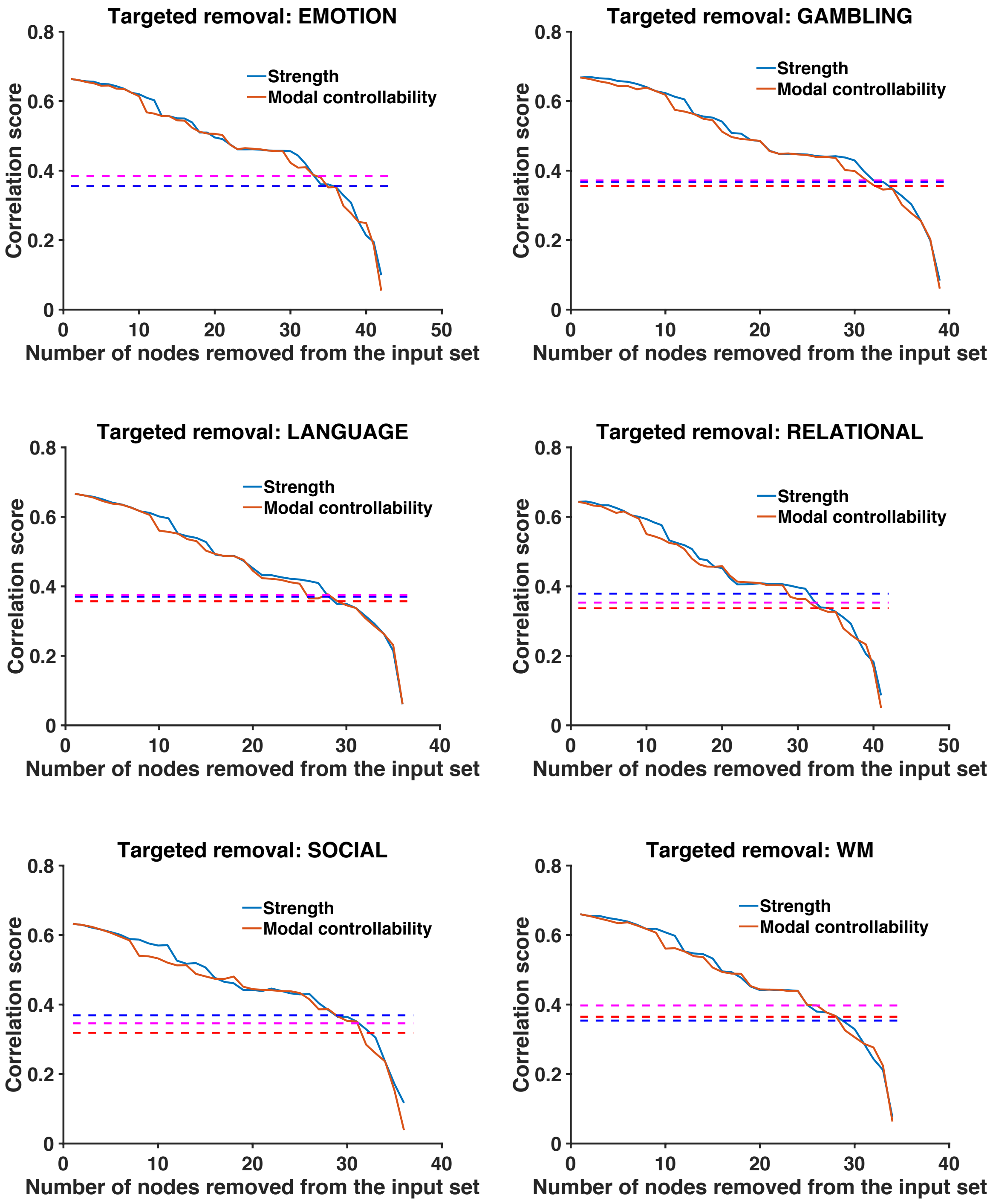

Figure S10: Robustness analysis. Evolution of the correlation between structure-informed $\mathbf{F}_{SI}$ and empirical functional connectivity $\mathbf{F}_{emp}$ as a function of the number of ROIs removed from the consensus input set. Dashed lines represent the three baselines, i.e. the correlation between $\mathbf{F}_{emp}$ and (i) the adjacency matrix of the connectome, (ii) $\mathbf{F}_{SI}$ based on a re-labelled connectome and (iii) $\mathbf{F}_{SI}$ obtained with a random input set. The removal ordering is fixed either by increasing weighted degree or decreasing model controllability.

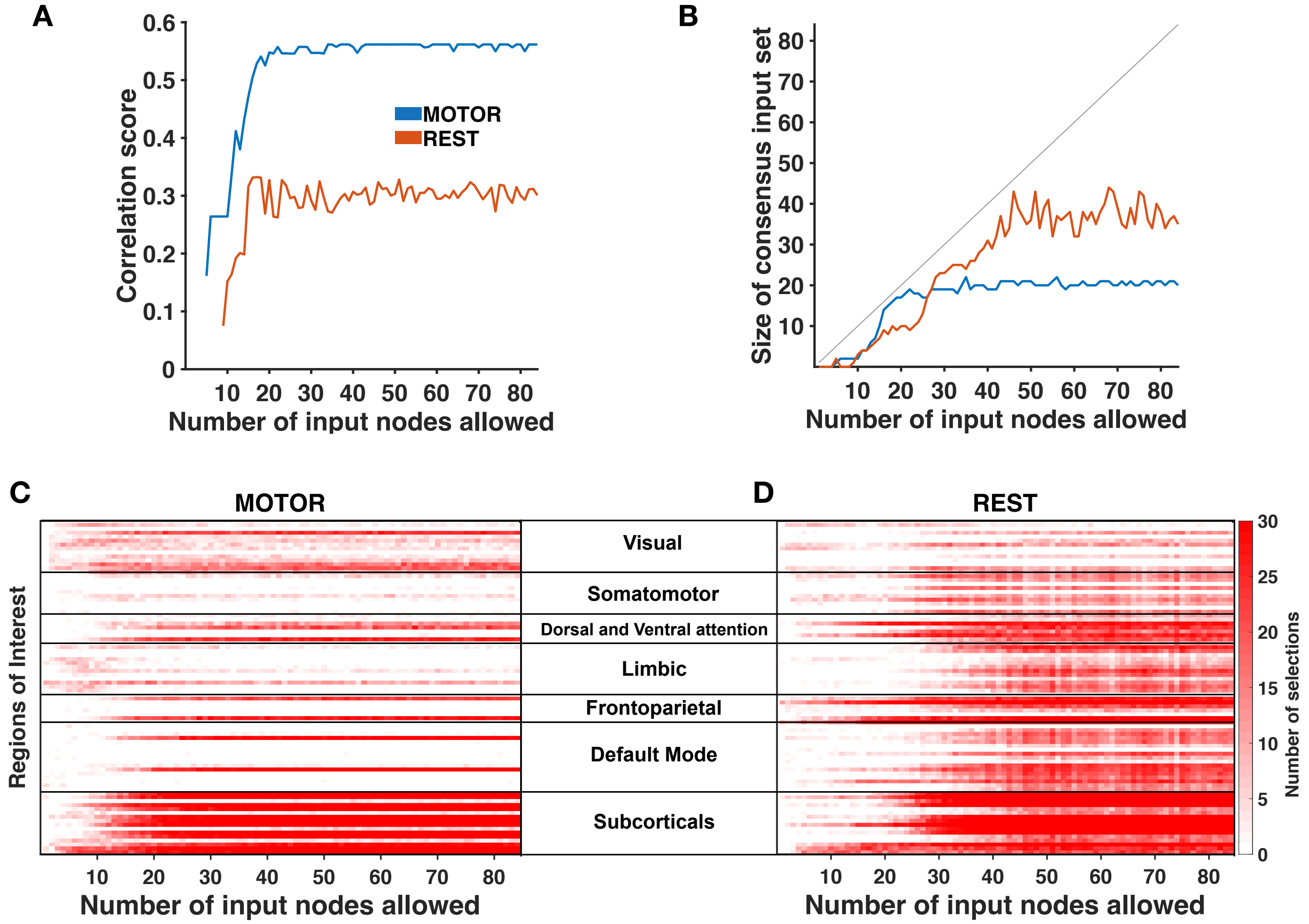

Figure S11: **Effect of brain parcellation.** **A**, **B**, **C** and **D** respectively reproduce the results presented in Figures 2A, 2B, 3A and 3B of the main text (motor task and resting-state only), when using the Desikan-Killiany atlas (84 ROIs).

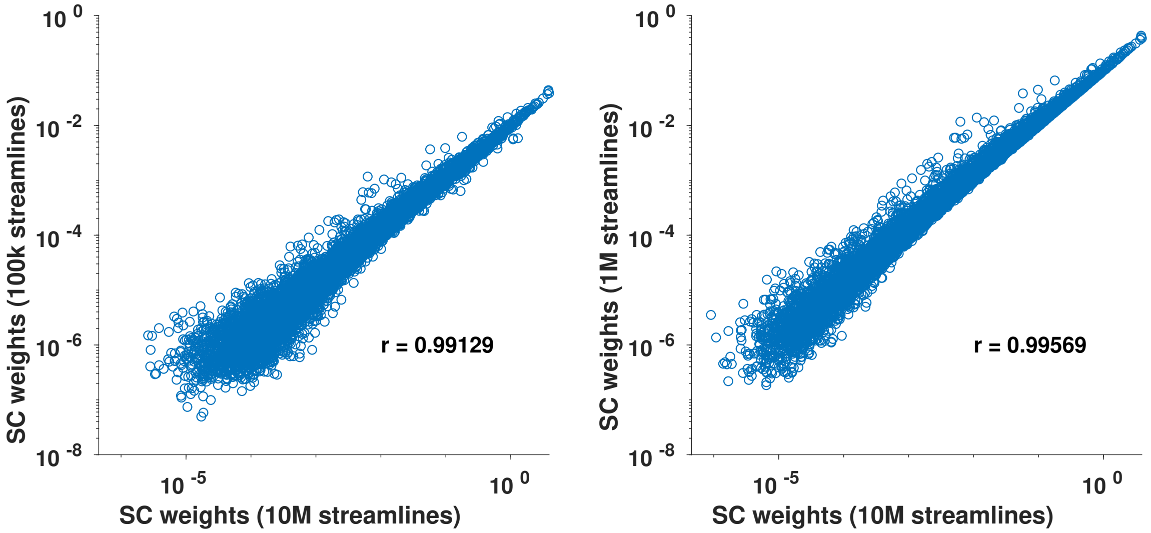

Figure S12: Comparison of structural connectivity (SC) values of the group-level connectomes derived from 10^5- and 10^6-streamlines-tractograms, with respect to that of the connectome used in the main analysis (10^7 streamlines).

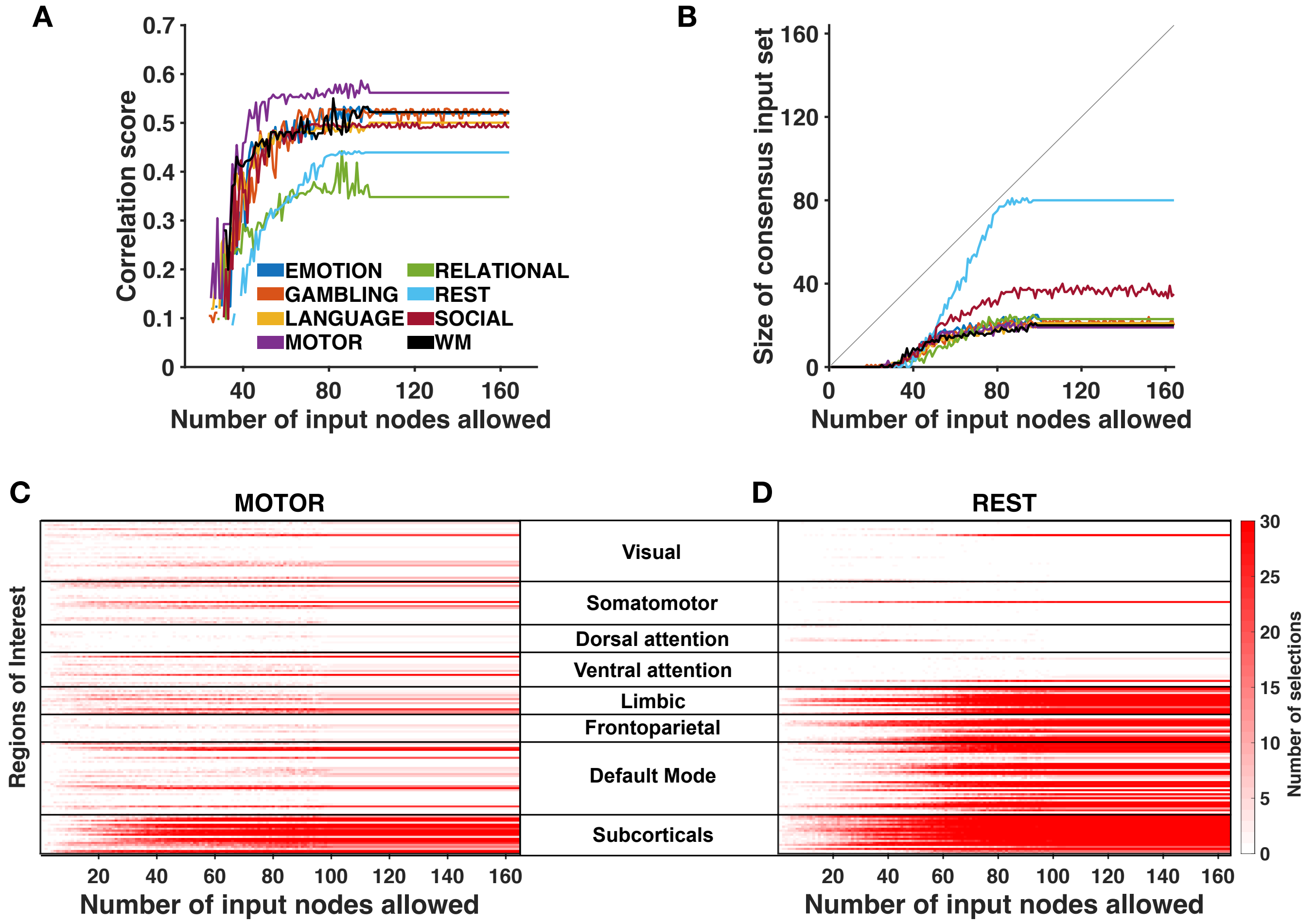

Figure S13: **Effect of Global Signal Regression on the main results.** **A**, **B**, **C** and **D** respectively reproduce the results presented in Figures 2A, 2B, 3A and 3B of the main text, when regressing out the global signal from the functional data.

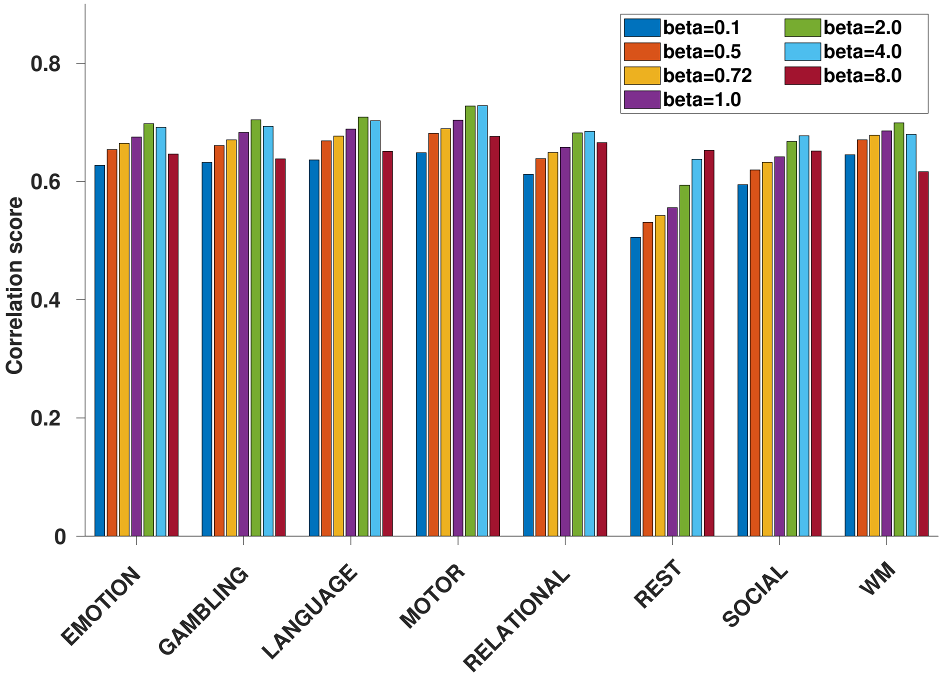

Figure S14: Comparison of optimized correlation scores for different values of β.

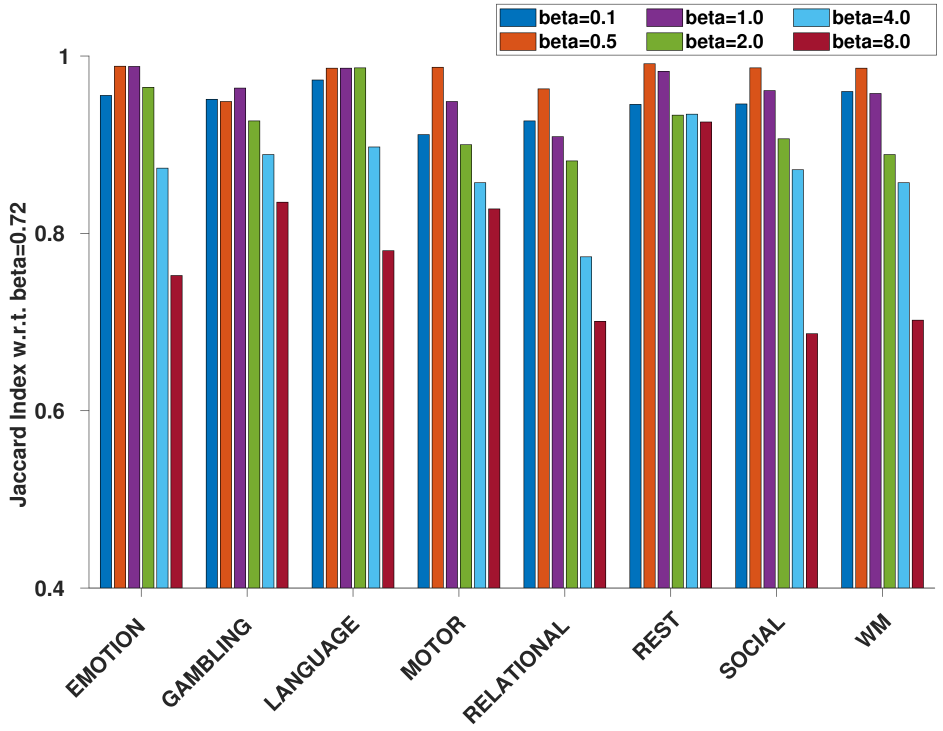

Figure S15: Jaccard index between the consensus input set (threshold: 25 selections over 30 runs) obtained with $\beta = 0.72$ and the consensus input sets obtained when varying β.

Supplementary Tables

Table S16: Comparison between three linear dynamics : the normalized Laplacian diffusion dynamics used in the main manuscript (DIFF), the dynamics defined by fixing the transition matrix $\mathbf{A}$ to the adjacency matrix of the connectome (ADJ) and the random-walk Laplacian diffusion dynamics (RW). In all cases, we performed 100 runs of the optimization algorithm, with $U = N$. The Jaccard index J is computed between the consensus input sets ($\geq$ 90 selections).

	EMO	GAM	LAN	MOT	REL	REST	SOC	WM
Mean correlation score : DIFF	0.66	0.67	0.68	0.7	0.65	0.54	0.63	0.68
Mean correlation score : ADJ	0.7	0.7	0.69	0.71	0.7	0.62	0.68	0.69
Mean correlation score : RW	0.67	0.68	0.68	0.7	0.66	0.56	0.65	0.68
J(DIFF,ADJ)	0.92	0.88	0.88	0.87	0.92	0.86	0.77	0.93
J(DIFF,RW)	0.96	0.94	0.93	0.91	0.99	0.94	0.96	0.97

Table S17: ROI-by-ROI numerical results coming along with Figures 4 and 5 of the main manuscript.ROIs that are consistently selected at least 90 times over 100 runs for all functional states are highlighted in blue.

ID	Hemisphere	Region of Interest	Number of selections by task								Yeo System
			EMOTION	GAMBLING	LANGUAGE	MOTOR	RELATIONAL	REST	SOCIAL	WM	
1	Left	Fronto-marginal gyrus and sulcus	5	2	0	0	3	99	5	0	DMN
2	Left	Inferior occipital gyrus and sulcus	0	0	0	0	0	0	0	0	VIS
3	Left	Paracentral lobule and sulcus	92	99	97	97	99	11	90	97	SOM
4	Left	Subcentral gyrus (central operculum) and sulci	0	0	0	0	0	0	1	0	SOM
5	Left	Transverse frontopolar gyri and sulci	97	28	0	53	86	100	100	2	DMN
6	Left	Anterior part of the cingulate gyrus and sulcus (ACC)	1	0	0	2	8	97	2	0	DMN
7	Left	Middle-anterior part of the cingulate gyrus and sulcus (aMCC)	1	1	0	0	0	2	3	0	VA
8	Left	Middle-posterior part of the cingulate gyrus and sulcus (pMCC)	1	3	1	0	5	0	1	0	SOM
9	Left	Posterior-dorsal part of the cingulate gyrus (dPCC)	100	97	10	85	100	97	100	53	DMN
10	Left	Posterior-ventral part of the cingulate gyrus (vPCC)	100	100	100	100	99	96	100	100	DMN
11	Left	Cuneus	0	0	0	0	0	0	4	0	VIS
12	Left	Opercular part of the inferior frontal gyrus	0	0	0	0	0	0	0	0	VA
13	Left	Orbital part of the inferior frontal gyrus	97	86	80	84	75	99	26	54	DMN
14	Left	Triangular part of the inferior frontal gyrus	1	0	0	2	0	17	0	0	DMN
15	Left	Middle frontal gyrus	0	0	0	0	0	0	0	0	FP
16	Left	Superior frontal gyrus	0	0	0	0	0	0	0	0	DMN
17	Left	Long insular gyrus and central sulcus of the insula	100	100	86	98	100	2	100	100	VA
18	Left	Short insular gyri	9	6	1	2	8	5	22	2	VA
19	Left	Middle occipital gyrus	0	0	0	0	0	0	0	0	VIS
20	Left	Superior occipital gyrus	0	0	0	0	0	0	0	0	VIS
21	Left	Lateral occipito-temporal gyrus	0	0	0	1	0	0	0	0	VIS
22	Left	Lingual gyrus, ligual part of the medial occipito-temporal gyrus	0	0	0	0	0	0	0	0	VIS
23	Left	Parahippocampal gyrus, parahippocampal part of the medial occipito-temporal gyrus	100	100	100	99	100	98	100	99	SUB
24	Left	Orbital gyri	11	5	0	9	3	97	2	3	LIM
25	Left	Angular Gyri	0	1	0	0	0	39	0	0	DMN
26	Left	Supramarginal gyrus	0	0	0	0	0	1	0	0	VA
27	Left	Superior parietal lobule	0	0	0	0	0	0	0	0	DA
28	Left	Postcentral gyrus	0	0	0	0	0	0	0	0	SOM
29	Left	Precentral gyrus	0	0	0	0	0	0	0	0	SOM
30	Left	Precuneus	0	0	0	1	0	4	0	0	DMN
31	Left	Straight gyrus	100	100	100	100	100	100	100	100	LIM
32	Left	Subcallosal area	100	100	100	100	100	100	100	100	SUB

ID	Hemisphere	Region of Interest	EMOTION	GAMBLING	LANGUAGE	MOTOR	RELATIONAL	REST	SOCIAL	WM	Yeo System
33	Left	Anterior transverse temporal gyrus	23	18	37	4	32	0	19	19	SOM
34	Left	Lateral aspect of the superior temporal gyrus	1	1	8	1	0	0	0	0	SOM
35	Left	Planum polare of the superior temporal gyrus	99	100	100	100	100	71	100	99	LIM
36	Left	Planum temporale or temporal plane of the superior temporal gyrus	0	1	0	0	2	0	0	0	VA
37	Left	Inferior temporal gyrus	0	0	0	1	0	22	0	0	LIM
38	Left	Middle temporal gyrus	0	0	0	0	0	4	0	0	DMN
39	Left	Horizontal ramus of the anterior segment of the lateral sulcus	64	29	26	21	25	97	10	15	DMN
40	Left	Vertical ramus of the anterior segment of the lateral sulcus	36	15	6	11	9	99	8	3	DMN
41	Left	Posterior ramus of the lateral sulcus	1	1	0	0	3	0	0	1	SOM
42	Left	Occipital pole	0	0	5	7	0	100	0	0	VIS
43	Left	Temporal pole	100	100	100	100	100	99	84	76	LIM
44	Left	Calcarine sulcus	0	0	0	0	0	0	0	0	VIS
45	Left	Central sulcus	0	0	0	0	0	0	0	0	SOM
46	Left	Marginal branch of the cingulate sulcus	1	0	0	1	3	0	0	2	VA
47	Left	Anterior segment of the circular sulcus of the insula	99	94	7	88	89	100	98	77	DMN
48	Left	Inferior segment of the circular sulcus of the insula	3	16	3	1	13	0	14	3	VA
49	Left	Superior segment of the circular sulcus of the insula	1	1	0	0	0	0	1	0	VA
50	Left	Anterior transverse collateral sulcus	100	100	100	100	50	100	50	100	LIM
51	Left	Posterior transverse collateral sulcus	0	3	85	82	0	0	0	68	VIS
52	Left	Inferior frontal sulcus	0	0	0	0	0	0	0	0	FP
53	Left	Middle frontal sulcus	0	0	0	0	0	65	0	0	FP
54	Left	Superior frontal sulcus	0	0	0	0	0	20	0	0	DMN
55	Left	Sulcus intermedius primus	99	84	3	4	95	99	34	0	DMN
56	Left	Intraparietal sulcus and transverse parietal sulci	0	0	0	0	0	0	0	0	DA
57	Left	Middle occipital sulcus and lunatus sulcus	0	0	0	0	0	0	0	0	VIS
58	Left	Superior occipital sulcus and transverse occipital sulcus	0	0	0	0	0	0	0	0	VIS
59	Left	Anterior occipital sulcus and preoccipital notch	0	0	1	1	0	0	0	0	VIS
60	Left	Lateral occipito-temporal sulcus	1	0	1	0	0	0	0	0	DA
61	Left	Medial occipito-temporal sulcus and lingual sulcus	1	2	13	8	0	0	0	1	VIS
62	Left	Lateral orbital sulcus	6	2	0	2	0	100	2	0	FP
63	Left	Medial orbital sulcus	100	100	100	100	100	100	100	100	LIM
64	Left	Orbital sulci	26	3	1	62	67	100	94	9	FP
65	Left	Parieto-occipital sulcus	0	0	0	0	0	0	83	0	VIS
66	Left	Pericallosal sulcus	100	100	95	100	100	100	100	98	DMN

ID	Hemisphere	Region of Interest	EMOTION	GAMBLING	LANGUAGE	MOTOR	RELATIONAL	REST	SOCIAL	WM	Yeo System
67	Left	Postcentral sulcus	0	0	0	0	0	0	0	0	DA
68	Left	Inferior part of the precentral sulcus	0	1	0	0	0	1	0	0	DA
69	Left	Superior part of the precentral sulcus	0	0	0	0	0	0	0	0	DA
70	Left	Suborbital sulcus	100	99	100	100	100	100	100	98	LIM
71	Left	Subparietal sulcus	28	28	6	11	64	18	7	8	DMN
72	Left	Inferior temporal sulcus	0	0	0	0	0	12	0	0	DMN
73	Left	Superior temporal sulcus	0	0	2	0	0	4	0	0	DMN
74	Left	Transverse temporal sulcus	66	81	67	8	79	0	71	44	SOM
75	Left	Cerebellum	0	0	0	0	0	61	0	2	CER
76	Left	Thalamus	100	100	100	95	100	99	99	100	SUB
77	Left	Caudate	36	7	1	53	15	100	12	4	SUB
78	Left	Putamen	49	13	1	19	51	100	30	32	SUB
79	Left	Pallidum	100	100	100	100	100	100	100	100	SUB
80	Left	Hippocampus	100	100	100	100	100	100	100	100	SUB
81	Left	Amygdala	100	100	100	100	100	100	100	100	SUB
82	Left	Accumbens area	100	100	100	100	100	100	100	100	SUB
83	Right	Thalamus	99	100	100	100	99	100	97	100	SUB
84	Right	Caudate	24	6	7	20	11	100	8	12	SUB
85	Right	Putamen	86	61	30	51	92	100	68	83	SUB
86	Right	Pallidum	100	100	100	100	100	100	100	100	SUB
87	Right	Hippocampus	100	100	100	100	100	99	100	100	SUB
88	Right	Amygdala	100	100	100	100	100	100	100	100	SUB
89	Right	Accumbens area	100	100	100	100	100	100	100	100	SUB
90	Right	Fronto-marginal gyrus and sulcus	23	2	1	3	15	100	23	4	FP
91	Right	Inferior occipital gyrus and sulcus	0	0	0	0	0	0	0	1	VIS
92	Right	Paracentral lobule and sulcus	99	98	100	95	100	98	99	98	SOM
93	Right	Subcentral gyrus (central operculum) and sulci	1	6	0	1	59	0	1	3	SOM
94	Right	Transverse frontopolar gyri and sulci	10	1	1	0	4	100	9	0	DMN
95	Right	Anterior part of the cingulate gyrus and sulcus (ACC)	1	0	0	0	3	96	1	0	DMN
96	Right	Middle-anterior part of the cingulate gyrus and sulcus (aMCC)	1	1	0	0	1	1	2	0	VA
97	Right	Middle-posterior part of the cingulate gyrus and sulcus (pMCC)	0	2	0	0	14	0	1	1	SOM
98	Right	Posterior-dorsal part of the cingulate gyrus (dPCC)	100	99	4	31	100	98	100	21	DMN
99	Right	Posterior-ventral part of the cingulate gyrus (vPCC)	100	100	100	100	100	5	100	100	DMN
100	Right	Cuneus	0	0	0	0	0	0	1	0	VIS
101	Right	Opercular part of the inferior frontal gyrus	0	0	0	0	0	0	0	0	VA

ID	Hemisphere	Region of Interest	EMOTION	GAMBLING	LANGUAGE	MOTOR	RELATIONAL	REST	SOCIAL	WM	Yeo System
102	Right	Orbital part of the inferior frontal gyrus	90	80	100	96	97	100	29	95	DMN
103	Right	Triangular part of the inferior frontal gyrus	1	1	5	2	1	8	0	0	FP
104	Right	Middle frontal gyrus	0	0	0	0	0	63	0	0	FP
105	Right	Superior frontal gyrus	0	0	0	0	0	0	0	0	DMN
106	Right	Long insular gyrus and central sulcus of the insula	99	100	100	98	100	1	99	100	VA
107	Right	Short insular gyri	14	9	8	1	42	4	55	12	VA
108	Right	Middle occipital gyrus	0	0	0	0	0	0	0	0	VIS
109	Right	Superior occipital gyrus	0	0	0	0	0	0	0	0	VIS
110	Right	Lateral occipito-temporal gyrus	0	0	1	0	0	0	0	0	VIS
111	Right	Lingual gyrus, ligual part of the medial occipito-temporal gyrus	0	0	0	0	0	0	0	0	VIS
112	Right	Parahippocampal gyrus, parahippocampal part of the medial occipito-temporal gyrus	100	100	100	100	98	57	100	100	SUB
113	Right	Orbital gyri	32	15	4	16	35	97	11	35	LIM
114	Right	Angular Gyri	0	0	0	0	1	88	0	0	DMN
115	Right	Supramarginal gyrus	0	0	0	0	0	0	0	0	VA
116	Right	Superior parietal lobule	0	0	0	0	0	0	0	0	DA
117	Right	Postcentral gyrus	0	1	0	0	1	0	0	0	SOM
118	Right	Precentral gyrus	0	0	0	1	0	0	0	0	SOM
119	Right	Precuneus	0	0	0	1	0	0	0	0	DA
120	Right	Straight gyrus	100	100	99	99	99	100	100	99	LIM
121	Right	Subcallosal area	100	100	100	100	100	100	100	100	SUB
122	Right	Anterior transverse temporal gyrus	57	74	68	13	80	0	65	53	SOM
123	Right	Lateral aspect of the superior temporal gyrus	0	0	21	0	61	0	0	2	SOM
124	Right	Planum polare of the superior temporal gyrus	100	100	100	99	100	52	100	100	LIM
125	Right	Planum temporale or temporal plane of the superior temporal gyrus	0	1	0	0	1	0	0	2	SOM
126	Right	Inferior temporal gyrus	0	0	0	2	0	0	0	0	DA
127	Right	Middle temporal gyrus	0	0	0	1	4	27	0	0	DMN
128	Right	Horizontal ramus of the anterior segment of the lateral sulcus	38	22	42	12	73	95	14	20	VA
129	Right	Vertical ramus of theanterior segment of the lateral sulcus	24	21	19	15	28	99	14	21	DMN
130	Right	Posterior ramus of the lateral sulcus	2	6	2	0	68	0	2	4	SOM
131	Right	Occipital pole	0	0	0	0	0	0	0	0	VIS
132	Right	Temporal pole	99	100	98	100	100	100	68	100	LIM
133	Right	Calcarine sulcus	0	0	0	0	0	0	0	0	VIS
134	Right	Central sulcus	0	0	0	0	11	0	0	0	SOM
135	Right	Marginal branch of the cingulate sulcus	2	6	0	0	13	0	2	4	VA
136	Right	Anterior segment of thecircular sulcus of the insula	96	32	18	71	54	97	55	26	FP

ID	Hemisphere	Region of Interest	EMOTION	GAMBLING	LANGUAGE	MOTOR	RELATIONAL	REST	SOCIAL	WM	Yeo System
137	Right	Inferior segment of thecircular sulcus of the insula	6	19	2	2	66	0	22	16	SOM
138	Right	Superior segment of thecircular sulcus of the insula	1	3	2	1	4	1	8	1	VA
139	Right	Anterior transverse collateral sulcus	100	100	100	100	100	100	47	100	LIM
140	Right	Posterior transverse collateral sulcus	1	0	71	8	0	0	0	39	VIS
141	Right	Inferior frontal sulcus	0	0	0	0	0	0	0	0	FP
142	Right	Middle frontal sulcus	0	0	0	0	0	33	0	0	FP
143	Right	Superior frontal sulcus	0	0	0	0	0	15	0	0	DMN
144	Right	Sulcus intermedius primus	0	0	1	0	1	94	0	0	DMN
145	Right	Intraparietal sulcus and transverse parietal sulci	0	0	0	0	0	0	0	0	DA
146	Right	Middle occipital sulcus and lunatus sulcus	0	0	0	0	0	0	0	0	VIS
147	Right	Superior occipital sulcus and transverse occipital sulcus	0	0	0	0	0	0	0	0	VIS
148	Right	Anterior occipital sulcus and preoccipital notch	0	0	0	0	2	0	0	2	VIS
149	Right	Lateral occipito-temporal sulcus	1	0	7	4	0	0	0	3	VIS
150	Right	Medial occipito-temporal sulcus and lingual sulcus	4	10	27	28	1	0	0	48	VIS
151	Right	Lateral orbital sulcus	1	2	0	1	3	98	2	2	FP
152	Right	Medial orbital sulcus	100	100	100	100	100	100	100	100	LIM
153	Right	Orbital sulci	47	61	2	90	65	100	85	64	FP
154	Right	Parieto-occipital sulcus	0	0	0	0	1	0	73	0	VIS
155	Right	Pericallosal sulcus	100	100	99	99	100	100	100	100	DMN
156	Right	Postcentral sulcus	0	0	0	0	0	0	0	0	DA
157	Right	Inferior part of the precentral sulcus	0	0	0	0	0	0	0	0	DA
158	Right	Superior part of the precentral sulcus	0	0	0	0	0	0	0	0	DA
159	Right	Suborbital sulcus	100	100	100	100	100	100	100	100	DMN
160	Right	Subparietal sulcus	6	3	1	3	14	39	6	0	DMN
161	Right	Inferior temporal sulcus	1	0	0	0	0	89	0	0	DMN
162	Right	Superior temporal sulcus	0	0	0	0	0	1	0	0	DMN
163	Right	Transverse temporal sulcus	32	33	62	14	75	0	36	50	SOM
164	Right	Cerebellum	0	0	0	0	0	39	0	1	CER
